# NMDA receptor kinetics drive distinct routes to chaotic firing in pyramidal neurons

**DOI:** 10.3389/fncom.2026.1753444

**Published:** 2026-06-03

**Authors:** Mehdi Borjkhani, Hadi Borjkhani, Morteza A. Sharif, Fariba Bahrami, Mahyar Janahmadi

**Affiliations:** 1 International Centre for Translational Eye Research (ICTER), Institute of Physical Chemistry, Polish Academy of Sciences, Warsaw, Poland; 2 Institute of Physical Chemistry, Polish Academy of Sciences, Warsaw, Poland; 3 School of Engineering – Energy and Information, HTW Berlin – University of Applied Sciences, Berlin, Germany; 4 Optics and Laser Engineering Group, Faculty of Electrical Engineering, Urmia University of Technology, Urmia, Iran; 5 CIPCE, Motor Control and Computational Neuroscience Laboratory, School of ECE, College of Engineering, University of Tehran, Tehran, Iran; 6 Neuroscience Research Center and Department of Physiology, Medical School, Shahid Beheshti University of Medical Sciences, Tehran, Iran

**Keywords:** addiction, chaos theory, computational neuroscience, information theory, neuronal dynamics, NMDA receptors, synaptic plasticity, visual processing

## Abstract

**Introduction:**

Neuronal firing patterns emerge from complex interactions between intrinsic membrane properties and synaptic receptor dynamics. N-methyl-D-aspartate (NMDA) receptors critically shape calcium influx and synaptic plasticity through their voltage-dependent Mg^2+^ block and prolonged activation kinetics, yet how their closing kinetics interact with glutamatergic drive and GABAergic modulation to control neuronal dynamics and information processing remains incompletely understood.

**Methods:**

We developed a Hodgkin–Huxley-type computational model incorporating NMDA, AMPA, and GABA receptor kinetics to investigate how the NMDA receptor closing rate β_*NMDA*_ and glutamatergic stimulation frequency control neuronal dynamics. We performed a systematic analysis of over 2.9 million inter-spike intervals across a large multi-parameter sweep of NMDA kinetics, glutamatergic stimulation frequency, and GABAergic modulation. Dynamical behavior was characterized using entropy–Lyapunov correlation analysis and frequency-dependent bifurcation analysis, and CaMKII phosphorylation was quantified to link kinetic regimes to downstream plasticity signaling.

**Results:**

The analysis revealed two mechanistically distinct pathways to firing irregularity. Pathway 1 (rapid-deactivation irregularity) emerged under relatively fast NMDA deactivation combined with specific input-frequency conditions, producing deterministic chaos with compromised information encoding. Pathway 2 (prolonged-activation irregularity) resulted from slow NMDA deactivation under weak drive, creating irregularity through sustained receptor activation and calcium influx. An optimal kinetic window emerged at β_*NMDA*_ = 0.042 ms^−1^, maximizing information transfer (0.275 bits) while maintaining stable dynamics. Entropy–Lyapunov correlation analysis confirmed deterministic chaos, and frequency-dependent bifurcation analysis demonstrated progressive narrowing and displacement of chaotic windows across the analyzed β_*NMDA*_ range as stimulation frequency increased. GABAergic inhibition provided frequency-selective stabilization, expanding the stable parameter space by 34.2% while preserving gamma oscillations. CaMKII phosphorylation analysis revealed that prolonged NMDA activation maintained elevated phosphorylation levels, creating conditions for pathological long-term potentiation.

**Discussion:**

These findings establish NMDA receptor kinetics as fundamental controllers of cortical excitability and information processing. The dual-pathway framework provides mechanistic insights into addiction-related memory formation, where prolonged NMDA activation enables pathological plasticity, and into visual processing disorders, where altered kinetics disrupt retinal function and cortical oscillatory balance. The identification of optimal kinetic windows and frequency-selective GABA modulation suggests therapeutic strategies based on kinetically specific interventions for neuropsychiatric disorders involving NMDA dysfunction.

## 1 Introduction

Information processing in the brain depends on precisely tuned interactions between intrinsic membrane conductances and synaptic receptor dynamics. NMDA receptors play a pivotal role in excitatory synaptic transmission, distinguished by their voltage-dependent Mg^2+^ block and relatively slow deactivation kinetics (Kampa et al., 2004; Vargas-Caballero and Robinson, 2004). These characteristics mediate extended calcium influx into postsynaptic neurons, influencing long-term potentiation (LTP), long-term depression (LTD), and other plasticity-related processes (Lüscher and Malenka, 2012). Beyond synaptic plasticity, NMDA receptors modulate neuronal firing stability and variability, with direct consequences for information encoding and transmission (Harsch and Robinson, 2000; Hunt and Castillo, 2012).

A central question in computational neuroscience concerns how changes in NMDA receptor gating kinetics—particularly closing rates (β_*NMDA*_) and glutamate stimulation frequencies—transition neurons between stable periodic firing and irregular chaotic regimes (Durstewitz and Gabriel, 2007; Soudry and Meir, 2012). While irregular spiking can degrade information transfer reliability, it may also increase dynamic range and encoding flexibility (de Ruyter van Steveninck et al., 1997; Ermentrout et al., 2008). Despite extensive research on excitatory-inhibitory balance effects on neuronal dynamics, the detailed parameter space of NMDA receptor kinetics has received limited systematic investigation. The precise influence of NMDA receptor kinetics on plasticity induction, inter-spike interval (ISI) frequency band emergence, and their implications for single-cell information encoding remains incompletely understood.

The clinical significance of NMDA receptor kinetic alterations extends across multiple neuropsychiatric conditions. In schizophrenia, altered receptor kinetics may contribute to gamma oscillation abnormalities and cognitive deficits (Coyle, 2012; Gandal et al., 2012). Autism spectrum disorders involve NMDA-mediated excitation-inhibition imbalances affecting sensory processing and social cognition (Rubenstein and Merzenich, 2003; Lee et al., 2017). Alzheimer's disease features progressive NMDA receptor dysfunction correlating with synaptic loss and memory impairment (Wang and Reddy, 2017; Snyder et al., 2005; Hynd et al., 2004). Chronic pain conditions exhibit altered NMDA kinetics in spinal cord circuits, contributing to central sensitization (Latremoliere and Woolf, 2009; Zhuo, 2016).

In addiction neuroscience, chronic substance exposure alters NMDA receptor expression and kinetics, contributing to pathological synaptic plasticity underlying addiction-related memory formation and relapse vulnerability (Kalivas, 2009; Wolf, 2016). Pathological memory formation represents a critical mechanism underlying addiction persistence and relapse vulnerability. Unlike normal learning and memory, drug-induced memories exhibit abnormal persistence, resistance to extinction, and heightened salience that can trigger craving and relapse even after prolonged abstinence (Kalivas, 2009; Wolf, 2016). These pathological memories form through aberrant synaptic plasticity mechanisms involving dysregulated NMDA receptor function and downstream signaling cascades, particularly calcium-dependent processes such as CaMKII phosphorylation (Lüscher and Malenka, 2011; Pascoli et al., 2014).

Previous computational modeling has demonstrated that opioid exposure fundamentally alters synaptic plasticity mechanisms through disruption of calcium homeostasis and CaMKII phosphorylation dynamics (Borjkhani et al., 2018a,b). These studies revealed that chronic drug exposure shifts the balance between LTP and LTD, favoring persistent synaptic modifications that encode drug-associated memories. NMDA receptors serve as critical gatekeepers in this process, with their kinetic properties determining calcium influx magnitude and persistence. Our research group has previously developed computational frameworks demonstrating how opioids induce pathological memory formation through theta rhythm generation during chronic consumption (Borjkhani et al., 2018c), and how cocaine disrupts action potential generation by reducing potassium currents (Borjkhani et al., 2022). These studies established the foundation for investigating drug-induced alterations in neural dynamics and synaptic plasticity mechanisms.

Evidence suggests NMDA receptor antagonists can disrupt drug memory reconsolidation and reduce relapse rates in preclinical studies (Lee et al., 2006; Das et al., 2013). However, the precise relationship between NMDA receptor kinetic properties and pathological memory formation vs. maintenance remains incompletely characterized. This knowledge gap represents a barrier to developing targeted therapeutic interventions that could selectively disrupt maladaptive memories while preserving normal memory function.

In visual neuroscience, NMDA receptors are essential for retinal ganglion cell function and cortical visual processing (Manookin et al., 2010; Fox and Daw, 1992). Receptor dysfunction contributes to glaucoma, diabetic retinopathy, and cortical visual impairment through altered firing patterns that disrupt normal visual processing (Bai et al., 2013; Seki and Lipton, 2008). In primary visual cortex, NMDA receptors mediate orientation selectivity refinement, ocular dominance plasticity, and contrast adaptation mechanisms (Morishita and Hensch, 2008; Hensch, 2005). The kinetic properties of these receptors directly influence critical period timing and the capacity for experience-dependent plasticity throughout life (Hensch, 2005).

Recent evidence suggests altered NMDA kinetics contribute to visual processing deficits in neurodevelopmental disorders. In amblyopia, disrupted NMDA-dependent plasticity prevents normal binocular integration, while in autism spectrum disorders, altered excitation-inhibition balance affects visual motion processing and gamma oscillations (Foss-Feig et al., 2013; Robertson and Baron-Cohen, 2017). Age-related changes in NMDA receptor function may contribute to declining visual processing efficiency in older adults, with reduced NMDA-mediated plasticity affecting contrast sensitivity and temporal processing (Hua et al., 2006).

Here, we develop and analyze a single-compartment Hodgkin-Huxley-type model of a pyramidal neuron incorporating sodium, potassium, and calcium currents, along with GABAergic, AMPA, and NMDA synaptic conductances. Building upon our previous computational investigations of opioid-induced memory formation (Borjkhani et al., 2018c) and cocaine effects on neuronal excitability (Borjkhani et al., 2022), we extend this framework to systematically examine NMDA receptor kinetic control of cortical dynamics. We include CaMKII phosphorylation dynamics as a biochemical pathway linking calcium transients to synaptic plasticity. By systematically varying β_*NMDA*_ and glutamatergic drive frequency, we evaluate how neuronal firing evolves through different oscillatory modes and assess impacts on spike-timing entropy, maximum Lyapunov exponent, and mutual information. We investigate how GABAergic inhibition modulates these transitions through fast inhibitory currents that control neuronal excitability and chaotic dynamics.

Our approach does not propose a new intrinsic pyramidal neuron model; rather, we adapt a well-established conductance-based framework (Golomb et al., 2006) and introduce a mechanism-focused synaptic-kinetics extension together with a systematic parameter-space analysis. The key innovation lies in treating the NMDA closing/deactivation rate (β_NMDA_) as the primary mechanistic control parameter and jointly sweeping it with stimulation frequency to construct a comprehensive regime map of spike-timing dynamics. This combination—kinetic control of NMDA decay via β_NMDA_, joint sweep with stimulation frequency, and integrated dynamical plus information-theoretic analysis—goes beyond prior single-cell pyramidal modeling studies that typically focus on intrinsic conductances or fixed synaptic time constants and do not provide a β_NMDA_× frequency regime map (Durstewitz and Gabriel, 2007; Harsch and Robinson, 2000). The physiological relevance of varying NMDA kinetics is supported by electrophysiological measurements showing that NMDA receptor Mg^2+^ unblock dynamics include fast and slow components that influence temporal integration and spike generation in cortical pyramidal neurons (Vargas-Caballero and Robinson, 2003, 2004; Kampa et al., 2004). Our findings reveal dual pathways to firing irregularity and establish NMDA receptor kinetics as fundamental controllers of cortical excitability and information processing. These results provide mechanistic insights into addiction-related memory disorders and visual processing dysfunction, suggesting therapeutic strategies targeting NMDA receptor kinetic normalization and oscillatory pattern restoration.

## 2 Materials and methods

**Figure 1:**
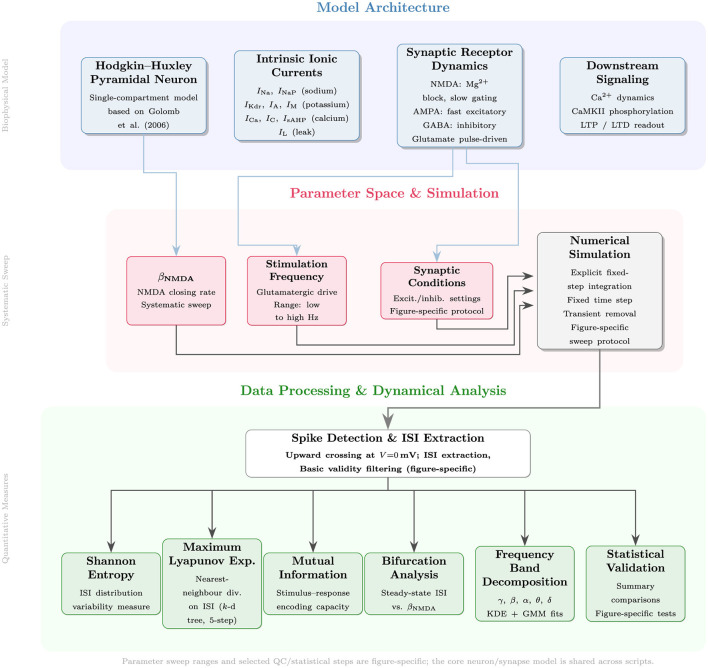
Computational workflow for analyzing NMDA kinetics and neuronal dynamics. The workflow summarizes the analysis pipeline used across figure-specific simulation scripts. *Model architecture:* a single-compartment Hodgkin–Huxley-type pyramidal neuron (Golomb et al.-based) with intrinsic ionic currents, synaptic receptor dynamics (NMDA/AMPA/GABA), and downstream Ca^2+^/CaMKII-related signaling modules. *Parameter space & simulation:* systematic sweeps of the NMDA closing-rate parameter (β_NMDA_) and stimulation frequency, with figure-specific synaptic conditions, using explicit fixed-step numerical integration, fixed time step, and transient removal. *Data processing:* spikes are detected by upward crossing at *V* = 0 mV, followed by inter-spike interval (ISI) extraction and basic figure-specific validity filtering. *Dynamical analysis:* ISI-based metrics and analyses including Shannon entropy, nearest-neighbor divergence-based maximum Lyapunov exponent (MLE)-like estimation (KD-tree implementation), mutual information, bifurcation analysis vs. β_NMDA_, frequency-band decomposition (KDE/GMM-based), and figure-specific statistical comparisons/tests. Parameter sweep ranges and some QC/statistical steps vary by script, while the core neuron/synapse model is shared across analyses.

Figure 1 provides an overview of our comprehensive computational approach to investigating NMDA receptor kinetics and their control over neuronal dynamics. The workflow encompasses four integrated stages: systematic model development and parameter space exploration, application of multiple dynamical analysis methods, identification of key mechanistic findings through the dual pathways framework, and translation to clinical applications. This systematic approach allows us to bridge molecular receptor properties with network-level dynamics and establish direct relevance to addiction neuroscience and visual processing disorders. The following sections detail each component of this workflow, beginning with the neuronal model architecture.

### 2.1 Neuronal model overview

**Figure 2:**
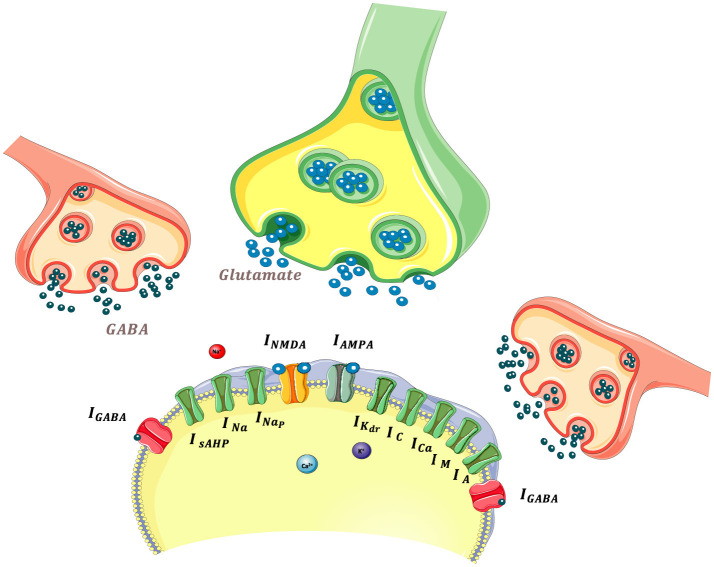
Synaptic inputs and ionic currents in the modeled neuron. This schematic illustrates the synaptic and intrinsic ionic currents included in the model. The total synaptic current consists of AMPA, NMDA, and GABAergic components, where AMPA and NMDA mediate excitatory transmission, with NMDA exhibiting a voltage-dependent Mg^2+^ block, while GABAergic currents provide inhibition. The intrinsic ionic currents include fast and persistent sodium (*I*_Na_, *I*_NaP_), various potassium currents regulating repolarization and adaptation (*I*_Kdr_, *I*_A_, *I*_M_), calcium-mediated currents (*I*_Ca_, *I*_C_), and slow after hyperpolarization and leak currents (*I*_sAHP_, *I*_L_). These currents collectively shape neuronal excitability, synaptic integration, and firing dynamics.

For the simulation of a pyramidal neuron in the cortex, we employed a single-compartment Hodgkin-Huxley-type model based on Golomb et al. (2006). The model was modified to incorporate glutamatergic and GABAergic receptors (NMDA, AMPA, and GABA receptors) alongside ionotropic channels, enabling responses to both excitatory and inhibitory neurotransmitters. Furthermore, the model incorporates CaMKII phosphorylation dynamics as a function of calcium concentration variations, providing a mechanistic link between synaptic activity and plasticity (Borjkhani et al., 2018a). This computational framework allows investigation of NMDA receptor kinetics effects on neuronal excitability relevant to addiction-related plasticity mechanisms and visual processing disorders. Figure 2 shows the general structure of the modeled elements.

#### 2.1.1 Model validation and verification

The model was validated against experimental data from pyramidal neurons in layers 2/3 of visual cortex Markram et al., (1997). Key validation metrics included: (1) resting potential (–65 ± 5 mV), (2) action potential amplitude (80–100 mV), (3) spike threshold (–45 ± 3 mV), and (4) adaptation ratio (0.6–0.8) during sustained current injection. Numerical integration was verified using analytical solutions for simplified cases and compared with NEURON simulator results (relative error < 0.1%).

### 2.2 Membrane dynamics

The membrane potential (*V*) in the excitatory postsynaptic neuron is governed by the following differential equation:


$$\begin{array}{l} C_{m}\frac{\partial V}{\partial t}=-(I_{\text{Na}}+I_{\text{NaP}}+I_{\text{Kdr}}+I_{\text{A}}+I_{\text{M}}+I_{\text{Ca}}+I_{\text{C}}+I_{\text{sAHP}}\\ \;+I_{\text{L}}+I_{\text{syn}}) \end{array}$$ (1)

where the membrane capacitance *C*_*m*_ = 1μF/cm^2^ and the total synaptic current is:


$$\begin{array}{l} I_{\text{syn}}=I_{\text{AMPA}}+I_{\text{NMDA}}+I_{\text{GABA}}\\ \;=g_{\text{AMPA}}m_{\text{AMPA}}\left(V-E_{\text{AMPA}}\right)\\ \;+g_{\text{NMDA}}m_{\text{NMDA}}B_{\text{Mg}}\left(V\right)\left(V-E_{\text{NMDA}}\right) \end{array}$$ (2)

$$\begin{array}{l} +g_{\text{GABA}}m_{\text{GABA}}\left(V-E_{\text{GABA}}\right) \end{array}$$ (3)

### 2.3 Intrinsic ionic currents

#### 2.3.1 Sodium currents

The transient sodium current is described by:


$$\begin{array}{l} I_{\text{Na}}=g_{\text{Na}}m^{3}h\left(V-E_{\text{Na}}\right) \end{array}$$ (4)

The activation and inactivation gating variables are denoted by *m* = *m*_∞_(*V*) and:


$$\begin{array}{l} \frac{dh}{dt}=\frac{h_{\infty }\left(V\right)-h}{\tau_{h}\left(V\right)} \end{array}$$ (5)

The time constant is:


$$\begin{array}{l} \tau_{h}\left(V\right)=0.1+0.75\cdot {\left[1+\exp\left(-\left(V+45\right)/\left(-6\right)\right)\right]}^{-1}\text{ms} \end{array}$$ (6)

The persistent sodium current is:


$$\begin{array}{l} I_{\text{NaP}}\left(V\right)=g_{\text{NaP}}\cdot p_{\infty }\left(V\right)\cdot \left(V-E_{\text{Na}}\right) \end{array}$$ (7)

#### 2.3.2 Potassium currents

The delayed rectifier potassium current is:


$$\begin{array}{l} I_{\text{Kdr}}\left(V,n\right)=g_{\text{Kdr}}n^{4}\left(V-E_{\text{K}}\right) \end{array}$$ (8)

The gating variable *n* follows:


$$\begin{array}{l} \frac{dn}{dt}=\frac{n_{\infty }\left(V\right)-n}{\tau_{n}\left(V\right)} \end{array}$$ (9)

with time constant:


$$\begin{array}{l} \tau_{n}\left(V\right)=0.1+0.5\cdot {\left[1+\exp\left(-\left(V+30\right)/\left(-15\right)\right)\right]}^{-1}\text{ms} \end{array}$$ (10)

The A-type potassium current has the following dynamics:


$$\begin{array}{l} I_{\text{A}}\left(V,b\right)=g_{\text{A}}\cdot a_{\infty }^{3}\left(V\right)\cdot b\cdot \left(V-E_{\text{K}}\right) \end{array}$$ (11)

where *a* = *a*_∞_(*V*) and:


$$\begin{array}{l} \frac{db}{dt}=\frac{b_{\infty }\left(V\right)-b}{\tau_{b}} \end{array}$$ (12)

The muscarinic-sensitive potassium current is:


$$\begin{array}{l} I_{\text{M}}\left(V,z\right)=g_{\text{M}}\cdot z\cdot \left(V-E_{\text{K}}\right) \end{array}$$ (13)

with gating variable dynamics:


$$\begin{array}{l} \frac{dz}{dt}=\frac{z_{\infty }\left(V\right)-z}{\tau_{z}} \end{array}$$ (14)

#### 2.3.3 Calcium currents

The high-voltage calcium current is:


$$\begin{array}{l} I_{\text{Ca}}\left(V,r\right)=g_{\text{Ca}}\cdot r^{2}\cdot \left(V-E_{\text{Ca}}\right) \end{array}$$ (15)

with gating variable:


$$\begin{array}{l} \frac{dr}{dt}=\frac{r_{\infty }\left(V\right)-r}{\tau_{r}} \end{array}$$ (16)

The fast calcium-activated potassium current is:


$$\begin{array}{l} I_{\text{C}}\left(V,c\right)=g_{\text{C}}\cdot d_{\infty }\left({\left[{\text{Ca}}^{2+}\right]}_{i}\right)\cdot c\cdot \left(V-E_{\text{K}}\right) \end{array}$$ (17)

where ${\left[{\text{Ca}}^{2+}\right]}_{i}$ is the intracellular calcium concentration, and:


$$\begin{array}{ll} \frac{dc}{dt} & =\frac{c_{\infty }\left(V\right)-c}{\tau_{c}} \end{array}$$ (18)

$$\begin{array}{ll} d_{\infty }\left({\left[{\text{Ca}}^{2+}\right]}_{i}\right) & ={\left[1+6/{\left[{\text{Ca}}^{2+}\right]}_{i}\right]}^{-1} \end{array}$$ (19)

The slow calcium-activated potassium current (afterhyperpolarization) is:


$$\begin{array}{l} I_{\text{sAHP}}\left(V,q\right)=g_{\text{sAHP}}\cdot q\cdot \left(V-E_{\text{K}}\right) \end{array}$$ (20)

with gating variable dynamics:


$$\begin{array}{ll} \frac{dq}{dt} & =\frac{q_{\infty }\left({\left[{\text{Ca}}^{2+}\right]}_{i}\right)-q}{\tau_{q}} \end{array}$$ (21)

$$\begin{array}{ll} q_{\infty }\left({\left[{\text{Ca}}^{2+}\right]}_{i}\right) & ={\left[1+2^{4}/{\left[{\text{Ca}}^{2+}\right]}_{i}^{4}\right]}^{-1} \end{array}$$ (22)

#### 2.3.4 Leak current

The leak current is modeled as:


$$\begin{array}{l} I_{\text{L}}=g_{\text{L}}\cdot \left(V-E_{\text{L}}\right) \end{array}$$ (23)

### 2.4 Calcium dynamics

The intracellular calcium concentration evolves according to:


$$\begin{array}{l} \frac{d{\left[{\text{Ca}}^{2+}\right]}_{i}}{dt}=-\alpha_{\text{Ca}}I_{\text{Ca}}-\alpha_{\text{NMDA, Ca}}I_{\text{NMDA}}-\alpha_{\text{AMPA, Ca}}I_{\text{AMPA}}\\ \;-\frac{{\left[{\text{Ca}}^{2+}\right]}_{i}}{\tau_{\text{Ca}}} \end{array}$$ (24)

where α_Ca_, α_NMDA, Ca_, and α_AMPA, Ca_ are calcium influx conversion factors and τ_Ca_ is the calcium extrusion/buffering time constant. All parameter values are provided in Supplementary Table S6.

### 2.5 Synaptic currents

#### 2.5.1 AMPA receptors

The AMPA-mediated current is calculated as:


$$\begin{array}{l} I_{\text{AMPA}}=g_{\text{AMPA}}\cdot m_{\text{AMPA}}\cdot \left(V-E_{\text{AMPA}}\right) \end{array}$$ (25)

The gating variable *m*_AMPA_ follows:


$$\begin{array}{l} \frac{dm_{\text{AMPA}}}{dt}=\alpha_{\text{AMPA}}\cdot G_{\text{glu}}\left(t\right)\cdot \left(1-m_{\text{AMPA}}\right)-\beta_{\text{AMPA}}\cdot m_{\text{AMPA}} \end{array}$$ (26)

where *G*_glu_(*t*) represents the time-varying glutamate concentration. All synaptic kinetic rate constants and conductance values are provided in Supplementary Table S4.

#### 2.5.2 NMDA receptors

The NMDA current is modeled as:


$$\begin{array}{l} I_{\text{NMDA}}=g_{\text{NMDA}}^{\text{eff}}\left(V,t\right)\cdot m_{\text{NMDA}}\cdot B_{\text{Mg}}\left(V\right)\cdot \left(V-E_{\text{NMDA}}\right) \end{array}$$ (27)

Here, $g_{\text{NMDA}}^{\text{eff}}\left(V,t\right)$ denotes the effective NMDA conductance, which in the implementation includes the baseline NMDA conductance and a fast auxiliary voltage-dependent modulation term. The voltage-dependent Mg^2+^ block is:


$$\begin{array}{l} B_{\text{Mg}}\left(V\right)=\frac{1}{1+\frac{{\left[{\text{Mg}}^{2+}\right]}_{o}}{3.75}\exp\left(-0.062V\right)} \end{array}$$ (28)

The NMDA receptor gating variable follows:


$$\begin{array}{l} \frac{dm_{\text{NMDA}}}{dt}=\alpha_{\text{NMDA}}\cdot G_{\text{glu}}\left(t\right)\cdot \left(1-m_{\text{NMDA}}\right)-\beta_{\text{NMDA}}\cdot m_{\text{NMDA}} \end{array}$$ (29)

where β_NMDA_ is the systematically varied closing rate (range: 0.002–0.1 ms^−1^). This parameter represents the key experimental variable in our study. The NMDA opening rate α_NMDA_ and all other receptor parameters are provided in Supplementary Table S4. **NMDA kinetic control parameter (β_NMDA_)**. The parameter β_NMDA_ governs the decay rate of the NMDA gating variable *m*_NMDA_: increasing β_NMDA_ accelerates NMDA current decay (shorter effective synaptic integration window, τ_NMDA_≈1/β_NMDA_), whereas decreasing β_NMDA_ prolongs NMDA activation (longer integration window). Importantly, β_NMDA_ modulates only the NMDA synaptic time course and does not alter intrinsic membrane conductances. We sweep β_NMDA_ over a physiologically motivated range (0.002–0.1 ms^−1^, corresponding to τ_NMDA_≈10–500 ms) and analyze how this kinetic parameter, together with stimulation frequency, controls the emergence of irregular spiking and dynamical transitions. This approach is supported by experimental work indicating that NMDA receptor dynamics and Mg^2+^ unblock exhibit both fast and slow components that affect spike generation and timing in cortical pyramidal neurons (Vargas-Caballero and Robinson, 2003, 2004; Kampa et al., 2004). Because NMDA kinetics strongly shape Ca^2+^ influx dynamics, β_NMDA_ is expected to affect downstream Ca^2+^/CaMKII signaling, consistent with computational work linking NMDA subunit-dependent kinetics to CaMKII activation thresholds (Santucci and Raghavachari, 2008).

#### 2.5.3 GABA receptors

The GABAergic inhibitory current is:


$$\begin{array}{l} I_{\text{GABA}}=g_{\text{GABA}}\cdot m_{\text{GABA}}\cdot \left(V-E_{\text{GABA}}\right) \end{array}$$ (30)

The GABA receptor gating variable follows:


$$\begin{array}{l} \frac{dm_{\text{GABA}}}{dt}=\alpha_{\text{GABA}}\cdot G_{\text{GABA}}\left(t\right)\cdot \left(1-m_{\text{GABA}}\right)-\beta_{\text{GABA}}\cdot m_{\text{GABA}} \end{array}$$ (31)

All ionic conductance densities, reversal potentials, synaptic kinetic rates, and gating variable parameters used in the simulations are listed in Supplementary Tables S2–S4.

### 2.6 Activation functions

All steady-state activation and inactivation functions follow the standard Boltzmann form:


$$\begin{array}{l} x_{\infty }\left(V\right)={\left[1+\exp\left(-\left(V-\theta_{x}\right)/\sigma_{x}\right)\right]}^{-1} \end{array}$$ (32)

where *x* can be replaced by *m*, *h*, *n*, *a*, *b*, *z*, *p*, *r*, or *c*. All gating variable parameters (θ_*x*_, σ_*x*_, and time constants) are provided in Supplementary Table S3. A complete specification of all model state variables, intrinsic ionic conductance parameters, synaptic receptor kinetics, CaMKII phosphorylation constants, initial conditions, and simulation parameters is provided in Supplementary Tables S1–S7.

### 2.7 CaMKII phosphorylation dynamics

Postsynaptic Ca^2+^ concentration variations lead to CaMKII phosphorylation, which is governed by equations adapted from Borjkhani et al. (2018a,b), and Zhabotinsky (2000):


$$\begin{array}{l} \text{Ph. CaMKII}=f_{\text{CaMKII}}\left({\left[{\text{Ca}}^{2+}\right]}_{i}\right) \end{array}$$ (33)

The detailed phosphorylation cascade involves 10 differential equations governing the concentrations of i-fold phosphorylated CaMKII (*P*_*i*_, where *i* = 0, 1, ..., 10). The system includes:

#### 2.7.1 Phosphorylation rates


$$\begin{array}{ll} v_{1} & =\frac{10k_{1}{\left(\left[{\text{Ca}}^{2+}\right]/K_{H1}\right)}^{8}P_{0}}{{\left(1+{\left(\left[{\text{Ca}}^{2+}\right]/K_{H1}\right)}^{4}\right)}^{2}} \end{array}$$ (34)

$$\begin{array}{ll} v_{2} & =\frac{k_{1}{\left(\left[{\text{Ca}}^{2+}\right]/K_{H1}\right)}^{4}}{1+{\left(\left[{\text{Ca}}^{2+}\right]/K_{H1}\right)}^{4}} \end{array}$$ (35)

$$\begin{array}{ll} v_{3} & =\frac{k_{2}e_{p}}{K_{M}+\sum_{i=1}^{10}iP_{i}} \end{array}$$ (36)

The total phosphorylated CaMKII is:


$$\begin{array}{l} \text{Ph. CaMKII}=f_{\text{CaMKII}}\left({\left[{\text{Ca}}^{2+}\right]}_{i}\right)=\overset{10}{\underset{i=1}{\sum }}P_{i} \end{array}$$ (37)

### 2.8 Computational implementation

#### 2.8.1 Software and hardware environment

All simulations and analyses used to generate results were implemented in Python (Spyder environment) on a Windows 11 Pro system using standard scientific Python libraries, including NumPy, SciPy, Matplotlib, scikit-learn, Seaborn, and Pandas. Data serialization and session storage were handled using pickle, dill, and joblib.

Computations were performed locally on a Dell Pro Max 16 Plus MB16250 laptop (64-bit Windows 11 Pro, x64-based processor) equipped with an Intel^®^ Core™ Ultra 9 285HX CPU (2.80 GHz), 64.0 GB RAM (63.5 GB usable; 6,400 MT/s), and 12 GB graphics memory. Simulations were not executed on a high-performance computing cluster.

#### 2.8.2 Numerical integration, simulation parameters, and parameter space exploration

Simulations were performed with a fixed time step of Δ*t* = 0.05 ms and total duration of 10 s (10,000 ms). The first 2 s (2,000 ms) of each simulation were discarded as transient prior to spike-train analysis. Stimulation was implemented as 5 ms rectangular pulses.

**Figure 3:**
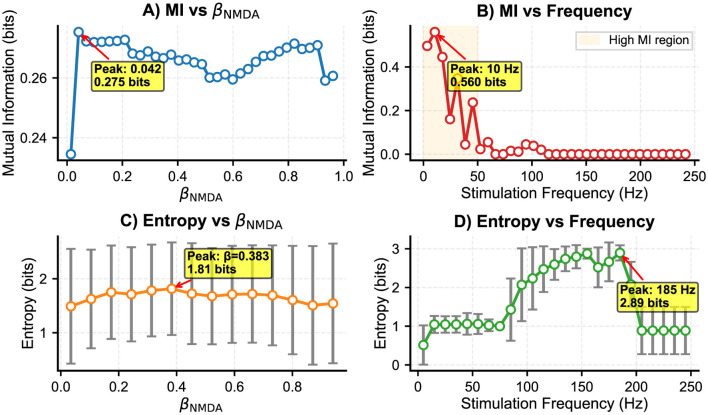
Information-theoretic analysis reveals optimal NMDA receptor modulation and frequency-dependent neural coding. **(A)** Stratified mutual information MI_*A*_(β_*i*_) = MI(*f*_stim_; ISI ∣ β_NMDA_ ∈ bin_*i*_), computed within each β_NMDA_ bin, showing how the ISI pattern encodes the stimulation frequency as the NMDA kinetic operating point is varied. Peak information transfer occurs at β_NMDA_ = 0.042 ms^−1^ (0.275 bits). **(B)** Stratified mutual information MI_*B*_(*f*_*j*_) = MI(β_NMDA_; ISI ∣ *f*_stim_ ∈ bin_*j*_), computed within each stimulation-frequency bin, showing how informatively the ISI distribution encodes β_NMDA_ across stimulation frequencies; encoding is highest in the low-frequency range (0–30 Hz, shaded region) and decays rapidly above ~50 Hz. **(C)** Neural response entropy as a function of β_NMDA_, showing that entropy increases with β_NMDA_ and approaches saturation at high values in the global scan. **(D)** Entropy exhibits frequency-dependent modulation with a prominent peak at 185 Hz (2.89 bits), followed by a sharp decline. Error bars represent standard deviation. Together, **(A–D)** indicate that moderate NMDA kinetic values optimize information transfer while maintaining response diversity, with distinct coding regimes for low-frequency information transmission and high-frequency response entropy. See Section 2.9.2 for the stratified MI definition.

Parameter exploration was conducted in two stages. First, a global two-dimensional sweep (used for Figure 3) was performed over β_NMDA_ and stimulation frequency to map broad trends in ISI-based dynamical and information-theoretic metrics. In this global sweep, stimulation frequency was sampled linearly from 0 to 245 Hz in 5 Hz increments, and β_NMDA_ was sampled linearly from 0 to 0.975 in increments of 0.025.

Second, a focused sweep was used for detailed dynamical visualization and bifurcation-style analyses within the physiologically relevant regime (β_NMDA_ ≤ 0.1 ms^−1^) and within stimulation-frequency ranges identified as highly informative by the global information-theoretic scan. GABAergic stimulation conditions were examined in these focused analyses, as described in the corresponding figure-specific sections.

Because different figures were generated using dedicated analysis scripts, the exact sampling grids (e.g., linear vs. logarithmic spacing), inclusion of GABAergic conditions, and replication/statistical procedures are specified in the relevant figure-specific methods and captions.

### 2.9 Data analysis pipeline

#### 2.9.1 Spike detection and quality control

Spikes were detected using an upward threshold-crossing criterion at 0 mV, i.e., events satisfying *V*(*t*_*i*−1_) < 0 and *V*(*t*_*i*_)≥0, and spike times were obtained from the corresponding crossing indices for subsequent ISI analysis.

#### 2.9.2 Dynamical analysis measures

##### 2.9.2.1 Shannon entropy

ISI variability was quantified using 20 equal-width bins based on Sturges' rule for the dataset size:


$$\begin{array}{l} H=-\overset{20}{\underset{k=1}{\sum }}p_{k}\log_{2}p_{k} \end{array}$$ (38)

where *p*_*k*_ represents the probability of the *k*-th ISI bin. Bin width was determined individually for each parameter combination to ensure adequate sampling.

##### 2.9.2.2 Maximum Lyapunov exponents

The maximum Lyapunov exponent (MLE) provides an objective dynamical-systems criterion for distinguishing deterministic chaos from merely variable spiking. The MLE quantifies the average exponential rate at which two initially nearby trajectories in state space diverge: MLE >0 indicates sensitive dependence on initial conditions and is a standard signature of chaotic dynamics; MLE ≈0 is consistent with periodic or quasi-periodic activity; and MLE < 0 indicates convergence toward stable dynamics. In this work, the MLE complements ISI-based bifurcation structure and entropy/MI metrics by providing an explicit, quantitative criterion for labeling chaotic parameter windows. The MLE is estimated using a nearest-neighbor divergence-based estimator applied to the ISI time series (KD-tree implementation), which serves as a computationally efficient MLE-like proxy for relative dynamical instability across parameter regimes; classical Lyapunov estimation methods such as (Wolf et al. 1985) provide the general methodological background. For each parameter setting, MLE was computed from the post-transient ISI time series (after discarding the initial 2,000 ms transient). Parameter points with MLE >0 were classified as chaotic and used to annotate the corresponding regions in bifurcation diagrams and heatmaps.

##### 2.9.2.3 Mutual information

In our single-neuron model, mutual information (MI) quantifies the statistical dependence between the parameter configuration driving the neuron and the resulting ISI distribution, following the established single-cell information-theoretic framework (Rieke et al., 1997; Borst and Theunissen, 1999): a neuron's spike train “encodes” information about its input parameters through the temporal structure of its firing pattern. For any two discrete variables *X* and *Y*, MI is defined as


$$\begin{array}{l} \text{MI}\left(X;Y\right)=\underset{x,y}{\sum }p\left(x,y\right)\log_{2}\frac{p\left(x,y\right)}{p\left(x\right)p\left(y\right)}, \end{array}$$ (39)

where *p*(*x, y*) is the joint distribution of bin indices and *p*(*x*), *p*(*y*) are the corresponding marginals.

MI was estimated from the pooled simulation data (one sample per observed ISI) using 35 equal-width bins for β_NMDA_, 35 bins for the stimulation frequency *f*_stim_, and 35 bins for the ISIs. Two complementary quantities were evaluated. First, a single global joint MI, MIβ_NMDA_, *f*_stim_);ISI, was computed over the full dataset to quantify the total amount of information about the two control parameters that is jointly encoded in the ISI distribution; by construction this yields a single scalar value and is not plotted in Figure 3. Second, to visualize how each control parameter individually modulates encoding capacity, we computed two stratified (within-bin) MI curves. For Figure 3A, the dataset was partitioned into bins of β_NMDA_, and within each bin *i* we computed MI_*A*_(β_*i*_) = MI*f*_stim_; ISI∣β_NMDA_ ∈ bin_*i*_, i.e., the MI between stimulation frequency and ISI using only samples whose β_NMDA_ falls in bin *i*. This curve reports how informatively the ISI pattern encodes *f*_stim_ at each β_NMDA_ operating point. For Figure 3B, the dataset was partitioned into bins of *f*_stim_, and within each bin *j* we computed MI_*B*_(*f*_*j*_) = MIβ_NMDA_; ISI∣*f*_stim_ ∈ bin_*j*_, giving the MI between the NMDA kinetic parameter and ISI at each stimulation-frequency operating point. Bins containing fewer than two samples, or only a single unique value of the other variable, were assigned MI = 0 (no estimable dependence). MI = 0 indicates that the ISI distribution carries no information about the conditioning variable, whereas higher values indicate that the firing pattern is more informative about it. The peak of MI_*A*_ at β_NMDA_ = 0.042 ms^−1^ (Figure 3A) therefore identifies the NMDA kinetic regime at which the neuron's spiking pattern is maximally informative about the stimulation frequency, representing an “optimal” encoding operating point.

#### 2.9.3 Frequency band classification

ISIs were categorized into physiologically relevant bands: gamma (7–33 ms, 30–100 Hz), beta (33–77 ms, 13–30 Hz), alpha (77–125 ms, 8–13 Hz), theta (125–250 ms, 4–8 Hz), and delta (250–2,000 ms, 0.5–4 Hz).

#### 2.9.4 Statistical analysis and validation

The main parameter sweeps were deterministic, and metrics at each parameter combination were computed directly from the corresponding simulated trajectory after transient removal. Results were primarily analyzed using grid-based visualization and descriptive comparisons (e.g., heatmaps, bifurcation-style plots, and parameter-response curves). Any figure-specific inferential tests or aggregation procedures are described in the corresponding methods or captions.

#### 2.9.5 Bifurcation diagram construction

**Figure 4:**
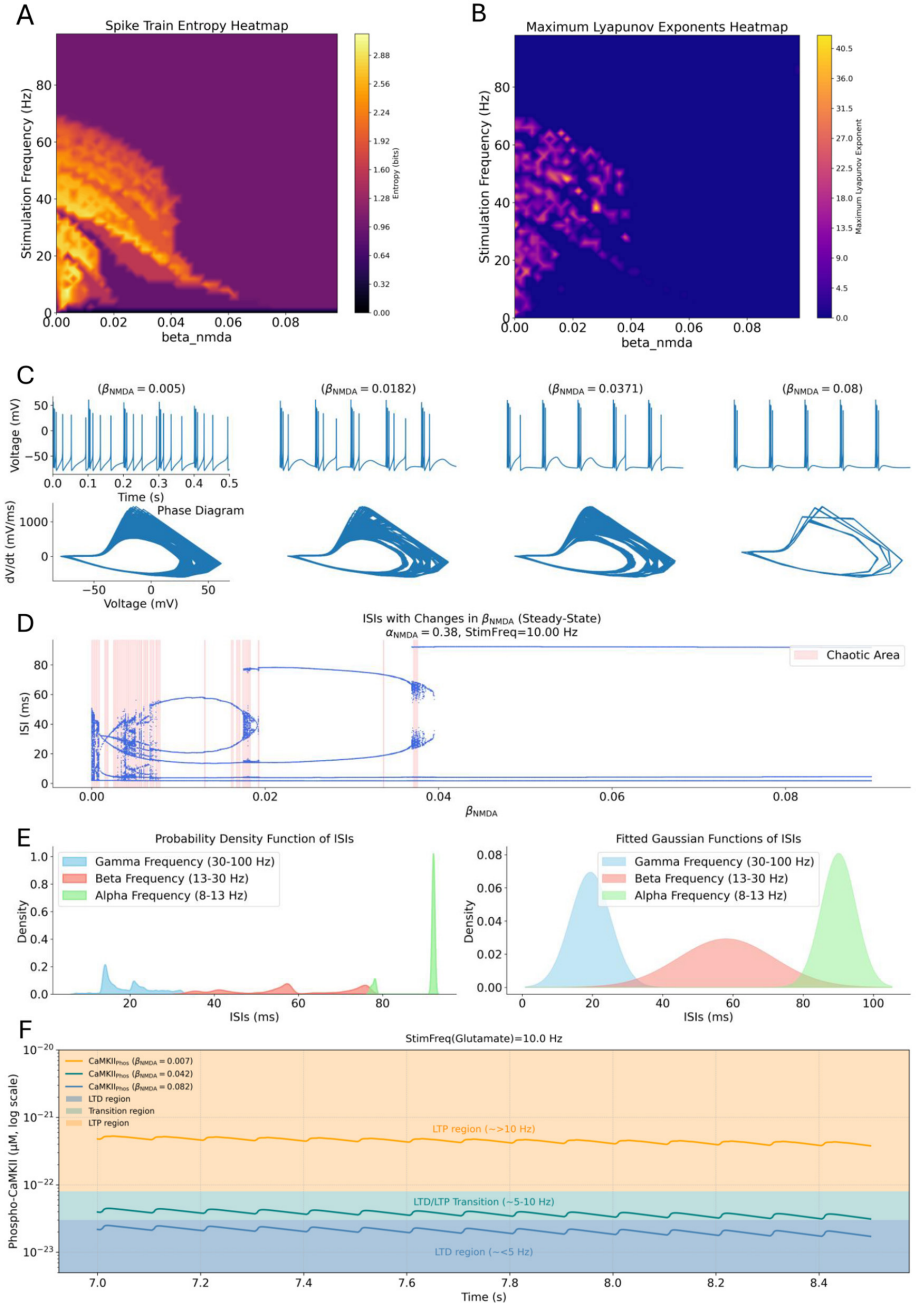
Bifurcation analysis and oscillatory dynamics. **(A, B)** Entropy and MLE heatmaps for low-frequency, low-β_NMDA_ parameter space. **(C)** Phase portraits at selected illustrative β_NMDA_ values demonstrating progressive evolution from regular periodic firing through period-doubling cascades to chaotic dynamics. **(D)** Bifurcation diagram revealing period-doubling route to chaos (pink-shadedregions indicate chaotic dynamics). **(E)** ISI spectral decomposition: left subpanel shows the empirical ISI probability density function (PDF) decomposed into physiological frequency bands (gamma 30–100 Hz, beta, alpha); right subpanel shows Gaussian-mixture fitted components used to quantify band contributions across β_NMDA_ conditions. **(F)** CaMKII phosphorylation trajectories across LTD, metaplasticity transition, and LTP regimes, linking dynamical transitions to downstream synaptic plasticity signaling.

**Figure 5:**
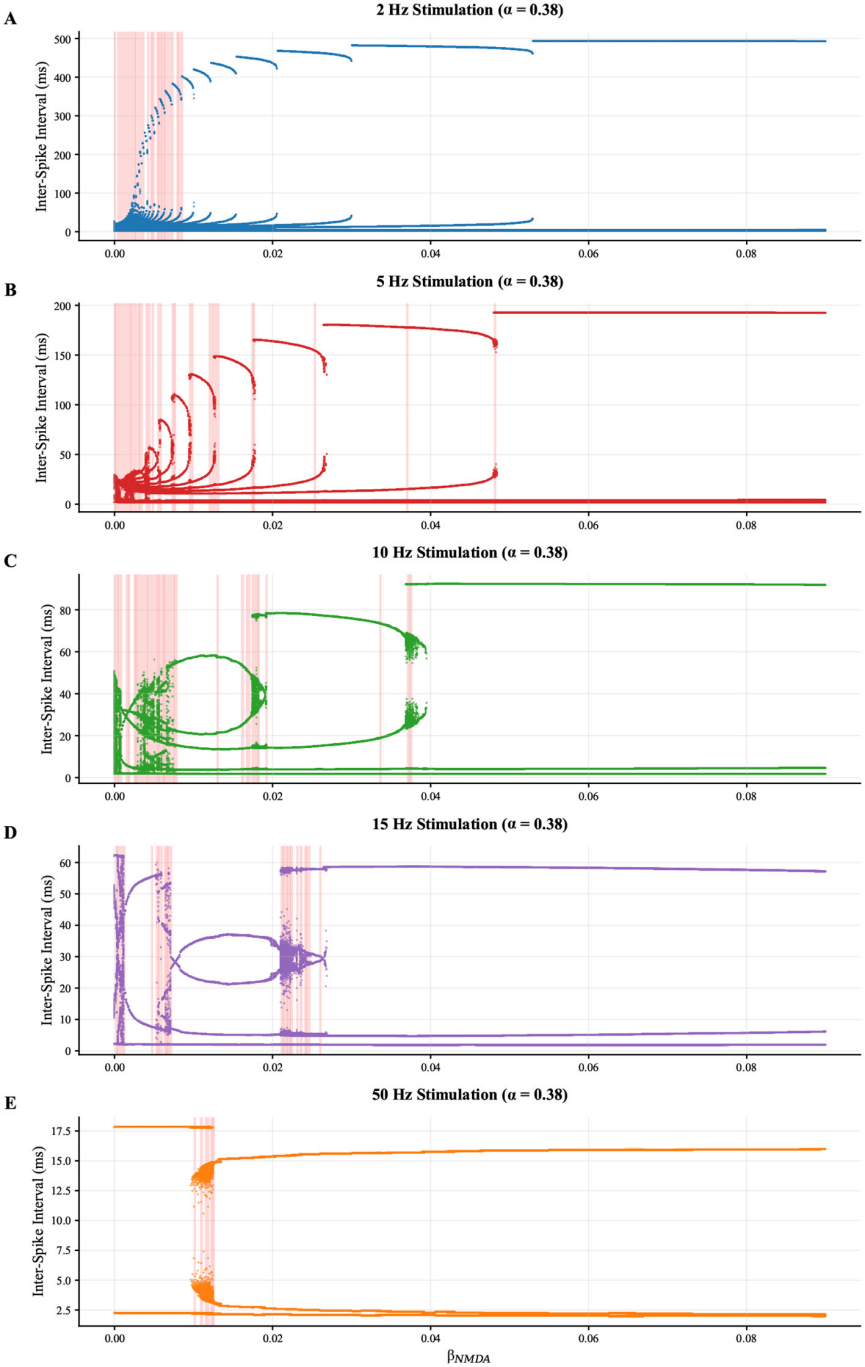
Frequency-dependent bifurcation landscapes and chaos onset thresholds. **(A–E)** Bifurcation diagrams showing inter-spike interval distributions vs. β_NMDA_ across stimulation frequencies from 2 Hz to 50 Hz. Panel titles report the fixed NMDA opening rate α_NMDA_ = 0.38 mM^−1^ms^−1^ used for each bifurcation slice. Pink shading indicates chaotic regions. Progressive frequency increases demonstrate systematic erosion of chaotic parameter windows: **(A)** 2 Hz exhibits complex bifurcations with broad chaotic regions across the β_NMDA_ sweep, **(B)** 5 Hz shows earlier destabilization with ISI compression, **(C)** 10 Hz displays narrowedchaotic windows, **(D)** 15 Hz demonstrates compressed parameter ranges shifted toward lower β_NMDA_ values, and **(E)** 50 Hz shows near-complete chaos eliminationwith tight gamma-frequency ISI clustering (15–18 ms).

Bifurcation diagrams were constructed from time-domain simulations rather than numerical continuation software (e.g., AUTO or XPPAUT). For each parameter setting (β_NMDA_, stimulation frequency, and GABAergic condition), the model was simulated for 10 s with time step Δ*t* = 0.05 ms. After discarding an initial 2,000 ms transient segment to ensure the system had reached its attractor, spike times were detected using the upward threshold-crossing criterion at 0 mV (Section 2.9.1), and inter-spike intervals (ISIs) were computed from consecutive spike times. The bifurcation diagram was generated by plotting the complete collection of post-transient ISIs against β_NMDA_ for a fixed stimulation frequency (scatter plot). In this representation, periodic regimes appear as single or finite sets of discrete branches (one ISI per cycle for period-1 orbits, or a small number of distinct ISIs for period-doubled orbits), period-doubling manifests as progressive branch splitting, and chaotic regimes appear as broadband, dense ISI distributions filling continuous intervals. To objectively label chaotic regions, we computed the maximum Lyapunov exponent (MLE) from the resulting ISI time series using the nearest-neighbor divergence-based estimator (KD-tree implementation) described in Section 2.9.2; classical Lyapunov estimation methods Wolf et al., (1985) provide the general methodological background; parameter points with MLE >0 were classified as chaotic and used to generate the pink-shaded chaos regions in bifurcation diagrams and the heatmaps in Figures 4, 5. All analyses were implemented in custom Python code and are available via the project repository.

### 2.10 Initial conditions robustness

Initial conditions for all state variables were chosen to approximate physiological resting values (Supplementary Table S6). To verify that model dynamics are independent of initial state selection, we performed two complementary sensitivity analyses. First, we constructed *V*–${\left[{\text{Ca}}^{2+}\right]}_{i}$ phase-plane trajectories for 25 combinations of initial membrane potential (*V*_0_ = −80 to −40 mV in 10 mV steps) and initial calcium concentration (${\left[{\text{Ca}}^{2+}\right]}_{i,0}=0$ to 2 μM in 0.5 μM steps). Under no-input conditions, all trajectories converged to a unique resting equilibrium, confirming a globally attracting fixed point (Supplementary Figure S1, left). Under glutamatergic stimulation, all initial conditions converged to the same limit cycle after the 2,000 ms transient discard period (Supplementary Figure S1, right). Second, we systematically varied synaptic gating initial conditions (*m*_AMPA_(0), *m*_NMDA_(0), *m*_GABA_(0)) from 0 to 1 and additionally performed 30 simulations with uniformly random initial gating variables. Steady-state firing rate was invariant across all conditions (40.25 ± 0.00 Hz, 0.0% variation), with differences confined to the initial transient period (Supplementary Figure S2). These results confirm that the reported bifurcation dynamics and ISI distributions are robust to initial condition selection.

### 2.11 Reproducibility statement

All simulation code and analysis scripts are available at https://github.com/borjkhani/Bifurcation_NMDA_FCN.

## 3 Results

**Figure 6:**
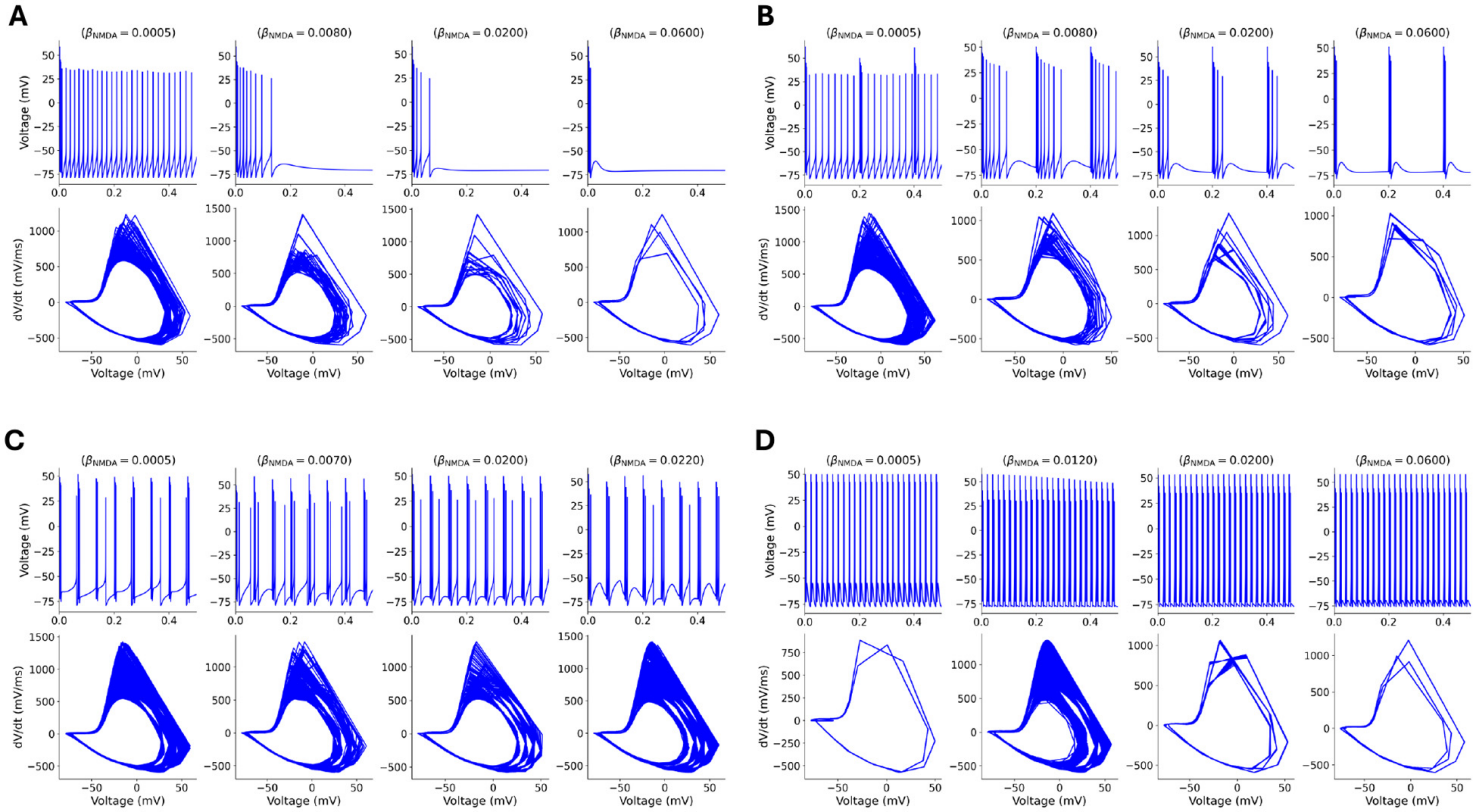
Neural dynamics across stimulation frequencies. Voltage traces and phase diagrams showing progressive frequency-dependent stabilization: **(A)** 2 Hz stimulation produces complex firing patterns with elaborate phase space structures including multi-loop attractors, **(B)** 5 Hz shows moderate parameter-dependent variability, **(C)** 15 Hz demonstrates further stabilization with constrained state space and simpler dynamical regimes, and **(D)** 50 Hz yields highly regular firing dominated by the driving frequency with tight limit cycles.

**Figure 7:**
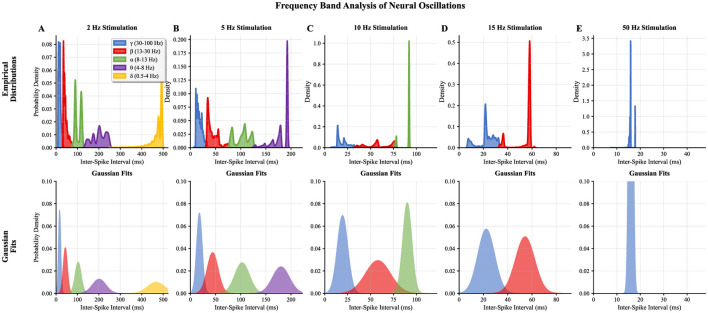
Frequency band analysis of neural oscillations across stimulation rates. **(A–E)** Top panels show empirical probability density distributions of inter-spike intervals decomposed into physiological frequency bands: gamma (30–100 Hz, blue), beta (13–30 Hz, red), alpha (8–13 Hz, green), theta (4–8 Hz, purple), and delta (0.5–4 Hz, yellow) across stimulation frequencies from 2 Hz to 50 Hz. Bottom panels display corresponding Gaussian mixture model fits. Progressive stimulation rate increases produce systematic spectral consolidation: **(A)** 2 Hz shows broad multi-band distributions, **(B–D)** intermediate frequencies (5–15 Hz) exhibit gradual gamma-band dominance, and **(E)** 50 Hz stimulation results in tight gamma-range concentration with minimal spectral diversity.

**Figure 8:**
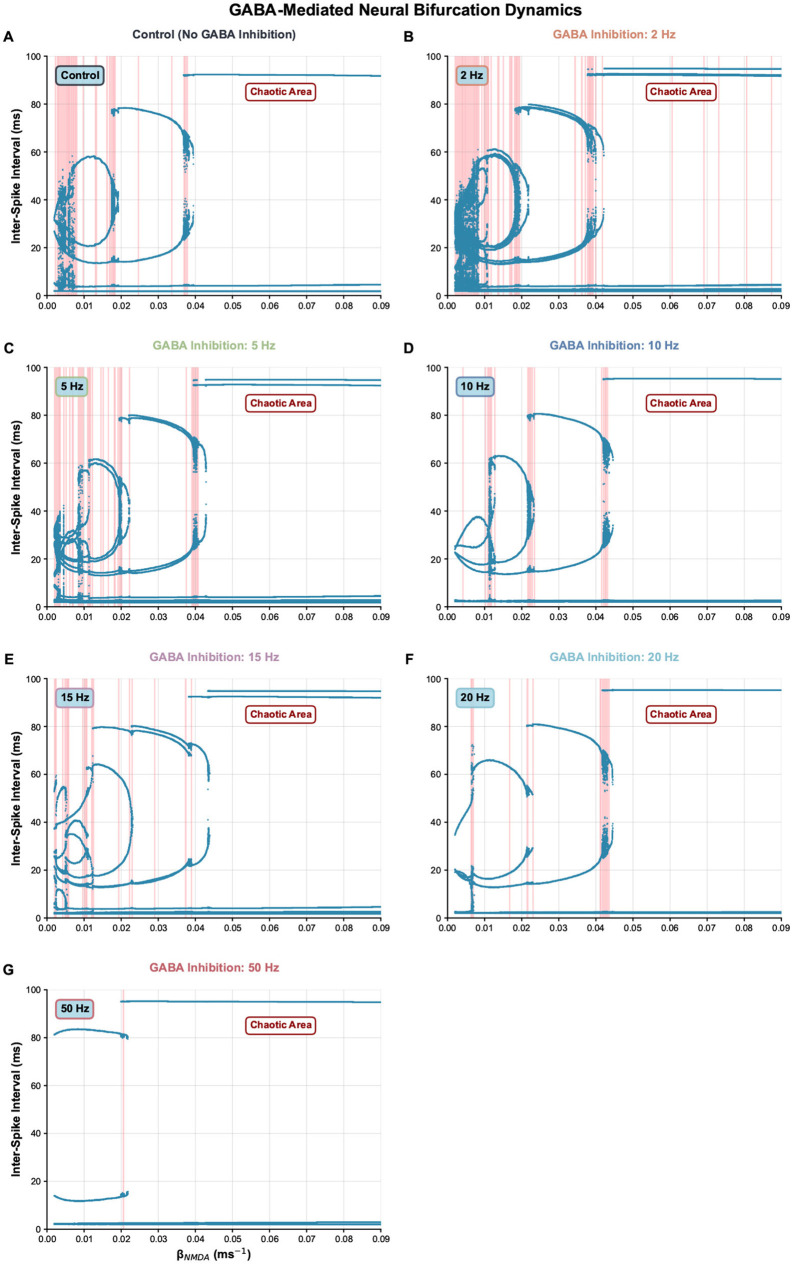
GABAergic inhibition modulates neural bifurcation dynamics. **(A–G)** Bifurcation diagrams showing inter-spike interval patterns vs. β_NMDA_ under increasing GABA inhibition frequencies: **(A)** 0 Hz control, **(B)** 2 Hz, **(C)** 5 Hz, **(D)** 10 Hz, **(E)** 15 Hz, **(F)** 20 Hz, and **(G)** 50 Hz. Pink shading indicates chaotic regions. GABA progressively stabilizes dynamics, with 50 Hz completely eliminating chaos.

**Figure 9:**
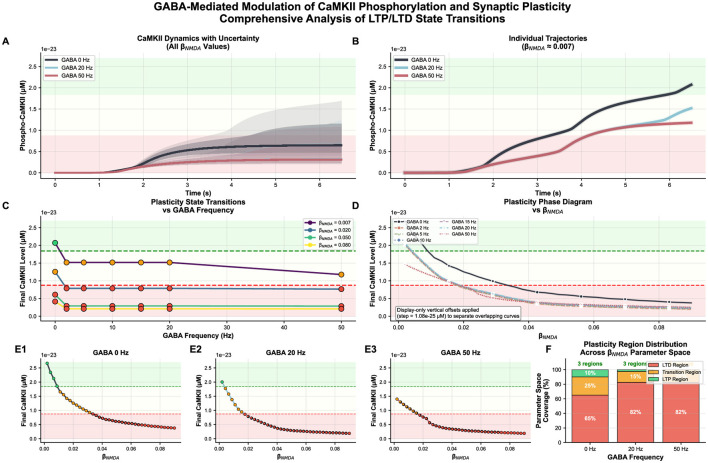
GABA-mediated modulation of CaMKII phosphorylation and synaptic plasticity. **(A)** CaMKII dynamics across all β_NMDA_ values with uncertainty bands for different GABA frequencies (0, 20, 50 Hz). **(B)** Individual CaMKII trajectories at β_NMDA_ = 0.007 ms^−1^ demonstrating GABA-dependent suppression. **(C)** Plasticity state transitions vs. GABA frequency for four β_NMDA_ values, with horizontal dashed lines indicating LTP/LTD thresholds. **(D)** Phase diagram of CaMKII levels vs. β_NMDA_ across GABA frequencies. **(E1–E3)** Detailed CaMKII trajectories for 0, 20, and 50 Hz GABA with plasticity-region color coding. **(F)** Parameter-space distribution showing a progressive shift from LTP-dominant (65%) to LTD-dominant (82%) states with increasing GABA inhibition. CaMKII is shown as a relative/normalized phosphorylation proxy (qualitative comparison only); plasticity regions/thresholds are heuristic model-defined regimes.

Results are organized in two stages: (i) a *global information-theoretic scan* across a broad β_NMDA_ and stimulation-frequency range to characterize entropy and mutual-information trends (Figure 3), and (ii) a *focused dynamical analysis* within the physiologically motivated β_NMDA_ = 0.002–0.1 ms^−1^ window and representative stimulation frequencies for bifurcation, phase-space, ISI-band, and plasticity analyses (Figures 4–9). Unless otherwise stated, quantitative summaries in Sections 3.3–3.8 refer to subsets of the parameter space selected for dynamical visualization within this focused window.

### 3.1 Model validation and basic neuronal response

**Figure 10:**
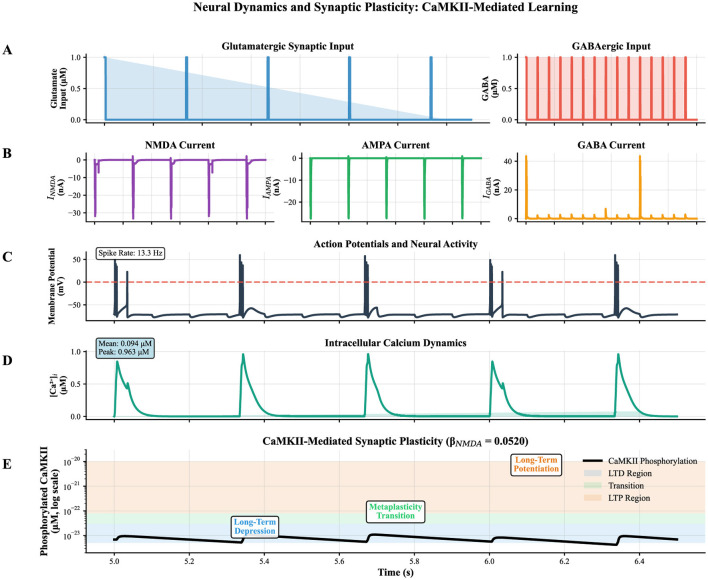
Neural dynamics and CaMKII-mediated synaptic plasticity under combined glutamatergic and inhibitory input (β_NMDA_ = 0.0520 ms^−1^). A representative 1.5 s post-transient window (5.0–6.5 s) illustrating the complete pipeline from synaptic input to plasticity signaling. **(A)** Presynaptic input patterns: glutamatergic rectangular pulses (1 μM; left) and pulsatile GABAergic input (1 μM; right). **(B)** Synaptic currents generated by the receptor models: NMDA current with slow sustained kinetics (peak ~30 nA), AMPA current with fast transient responses (peak ~25 nA), and GABA current providing inhibitory modulation (peak ~45 nA). **(C)** Postsynaptic membrane potential showing stimulus-evoked action potentials at 13.3 Hz; the dashed red line indicates the 0 mV spike detection threshold. **(D)** Intracellular Ca^2+^ concentration transients following synaptic activation and spiking (mean: 0.094 μM, peak: 0.963 μM). **(E)** CaMKII phosphorylation trajectory (logarithmic scale) plotted against the LTD (blue), metaplasticity transition (green), and LTP (orange) bands; under this stimulation condition, CaMKII remains within the LTD/transition range, indicating that the NMDA kinetics at this β_NMDA_ do not produce sustained Ca^2+^ influx sufficient to enter the LTP region.

The computational model reproduced characteristic pyramidal neuron dynamics under controlled stimulation conditions (Figure 10). Glutamatergic inputs (1 μM rectangular pulses) and pulsatile GABAergic inhibition (1 μM) generated distinct synaptic current profiles (Figures 10A–B). NMDA currents exhibited slow kinetics with sustained activation (peak ~30 nA), AMPA currents showed rapid transient responses (peak ~25 nA), and GABA currents provided pulsatile inhibitory modulation (peak ~45 nA). At β_NMDA_ = 0.0520 ms^−1^, the neuron fired action potentials at 13.3 Hz with membrane potential dynamics ranging from approximately −60 mV to +50 mV (Figure 10C). Intracellular calcium concentration ([Ca^2+^]_*i*_) exhibited spike-triggered transients reaching 0.963 μM peaks (mean: 0.094 μM; Figure 10D). CaMKII phosphorylation dynamics (Figure 10E), displayed on a logarithmic scale to resolve small but systematic activity-dependent modulations, demonstrated three distinct plasticity regimes: Long-Term Depression (LTD), metaplasticity transition zone, and Long-Term Potentiation (LTP) regions. Under this stimulation condition, CaMKII remained within the LTD/transition range, indicating that the NMDA kinetics and input pattern at this β_NMDA_ did not produce sustained calcium influx sufficient to drive phosphorylation into the LTP region. Figure 10 thus illustrates the complete mechanistic pipeline from synaptic stimulation to plasticity signaling: the timing of glutamatergic and inhibitory pulses (Figure 10A) determines the resulting NMDA, AMPA, and GABA synaptic currents (Figure 10B), which shape the postsynaptic spike output (Figure 10C). Spiking and synaptic activation generate intracellular Ca^2+^ transients (Figure 10D), which in turn drive CaMKII phosphorylation dynamics used to classify the learning state relative to LTD/transition/LTP thresholds (Figure 10E). This provides an interpretable bridge between synaptic kinetics (including β_NMDA_), spike timing, and CaMKII-mediated plasticity outcomes.

### 3.2 Information-theoretic analysis reveals optimal NMDA receptor modulation

Information-theoretic analysis identified optimal NMDA receptor kinetics for neural coding (Figure 3). Stratified mutual information between the stimulation frequency and the ISI pattern, computed within each β_NMDA_ bin (MI_*A*_; see Section 2.9.2), peaked at β_NMDA_ = 0.042 ms^−1^ with a maximum of 0.275 bits (Figure 3A), identifying a kinetic operating point at which the ISI pattern most informatively discriminates between input frequencies. Complementarily, stratified MI between β_NMDA_ and the ISI pattern, computed within each stimulation-frequency bin (MI_*B*_), showed the highest values in the low-frequency range (0–30 Hz) and rapid decay above 50 Hz (Figure 3B). Neural response entropy increased with β_NMDA_ and approached saturation at high β_NMDA_ values in the global information-theoretic scan (Figure 3C), indicating that ISI variability reaches a ceiling beyond which further changes in NMDA kinetics cannot increase firing irregularity. Entropy exhibited frequency-dependent modulation with a prominent peak at 185 Hz (Figure 3D). We focused the primary parameter sweep on β_NMDA_ ≤ 0.1 ms^−1^ (i.e., τ_NMDA_≥10 ms) because this range encompasses the physiologically realistic window of NMDA receptor deactivation kinetics. NMDA receptor-mediated currents are characterized by slow decay time constants (τ_decay_~50–300 ms for GluN2A/B-containing receptors), which persist well beyond the brief glutamate pulse and provide sustained depolarizing drive (Vargas-Caballero and Robinson, 2004; Kampa et al., 2004). This slow component is particularly important for temporal integration and calcium entry, since prolonged NMDA activation supports extended Ca^2+^ influx and thereby more effectively engages calcium-dependent signaling pathways such as CaMKII phosphorylation (Santucci and Raghavachari, 2008). Values at the lower end of our range (β_NMDA_≈0.002 ms^−1^, τ_NMDA_≈500 ms) correspond to the slowest physiological NMDA kinetics, while the upper boundary (β_NMDA_ = 0.1 ms^−1^, τ_NMDA_≈10 ms) represents fast-decaying NMDA components. The information-theoretic analyses in Figure 3 extend beyond this focused dynamical window to characterize global trends across a broader β_NMDA_ range; unless otherwise stated, quantitative summaries in Sections 3.3–3.8 refer to subsets of the parameter space selected for dynamical visualization and bifurcation analysis within the 0.002–0.1 ms^−1^ range.

### 3.3 Bifurcation analysis reveals period-doubling routes to chaos

Detailed bifurcation analysis in the low-frequency regime revealed systematic transitions in firing dynamics (Figure 4). Entropy and maximum Lyapunov exponent heatmaps across the β_NMDA_—frequency parameter space identified chaotic regions concentrated in low-frequency, low-β_NMDA_ parameter space, where the combination of slow NMDA deactivation and sparse synaptic drive produces the greatest dynamical complexity. Phase portraits at selected illustrative β_NMDA_ values (Figure 4C) demonstrated progressive evolution from regular periodic firing characterized by simple closed-loop limit cycles, through period-doubling cascades at intermediate values, to fully chaotic dynamics where trajectories filled extended regions of state space without repeating. These examples visualize trajectory morphologies at a fixed representative frequency and do not imply a globally monotonic dependence of chaos on β_NMDA_. Bifurcation diagrams constructed by plotting peak ISI values as a function of β_NMDA_ revealed classical period-doubling routes to chaos, with pink-shaded regions indicating chaotic parameter ranges identified by positive maximum Lyapunov exponents. Entropy-Lyapunov correlation analysis across the full parameter space confirmed the deterministic origin of the observed irregular dynamics, distinguishing the irregularity from stochastic noise.

Figures 4A–F summarizes the dynamical transition from regular firing to chaotic regimes across. Figures 4A, B show that regions of elevated Shannon entropy and positive maximum Lyapunov exponent co-localize in the parameter space, identifying windows where high spike-time unpredictability coincides with sensitive dependence on initial conditions—the hallmark of deterministic chaos. Figure 4C illustrates representative voltage traces and phase portraits across selected β_NMDA_ values, demonstrating the progression from periodic spiking (simple limit cycles) to period-doubled oscillations and irregular, space-filling attractors. Figure 4D shows the ISI-based bifurcation diagram: for each β_NMDA_ value, the complete set of post-transient ISIs is plotted, where single branches indicate periodic firing and progressive branch splitting indicates period-doubling; dense broadband ISI scatter corresponds to chaotic regimes. In Figure 4E, the left subpanel displays the empirical ISI probability density function (PDF) decomposed into physiological frequency bands (gamma, beta, and alpha bands based on ISI ranges), while the right subpanel shows Gaussian-mixture fitted components used to quantify band contributions consistently across β_NMDA_ conditions. Figure 4F links these dynamical regimes to synaptic plasticity signaling by showing CaMKII phosphorylation trajectories across LTD, metaplasticity transition, and LTP regimes, demonstrating how the transition to chaotic firing alters downstream plasticity-relevant calcium–CaMKII dynamics. Biologically, the chaotic regions correspond to parameter windows of high spike-time variability and reduced coding reliability, where the neuron's temporal precision is degraded and plasticity thresholds become destabilized. Because β_NMDA_ controls the effective NMDA integration window, the resulting stability landscape is not necessarily monotonic; instead, chaos occurs in discrete parameter windows separated by stable regions. This implies that NMDA kinetic dysregulation—arising from altered receptor subunit composition, modulatory state, or pharmacological intervention—should be interpreted as increased susceptibility to unstable firing regimes within specific β_NMDA_ intervals under physiologically relevant input frequencies, rather than a single monotonic disease threshold (Durstewitz and Gabriel, 2007). The non-monotonic appearance and reappearance of chaotic windows reflects the competing timescales of intrinsic membrane currents, NMDA synaptic decay, and calcium feedback that characterize the underlying nonlinear dynamics.

### 3.4 Frequency-dependent neural dynamics and phase space evolution

Voltage traces and phase diagrams demonstrated systematic changes in neural dynamics across stimulation frequencies (Figure 6). Figures 6A–D correspond to 2, 5, 15, and 50 Hz stimulation, respectively. At 2 Hz stimulation, neurons exhibited complex firing patterns with multiple β_NMDA_ values producing distinct voltage trajectories and elaborate phase space structures, including multi-loop attractors and aperiodic orbits. At 5 Hz stimulation (Figure 6B), the firing patterns showed earlier onset of irregular dynamics with moderate parameter-dependent variability. At 15 Hz (Figure 6C), further stabilization occurred as the increased synaptic drive constrained the system to simpler dynamical regimes with fewer coexisting attractors and reduced attractor dimensionality. At 50 Hz stimulation (Figure 6D), neuronal dynamics were dominated by the driving frequency, producing highly regular firing with simplified phase space orbits that converged to tight limit cycles regardless of β_NMDA_ value. This progressive frequency-dependent stabilization demonstrates that increasing synaptic drive systematically constrains the accessible state space, effectively overriding the intrinsic NMDA-dependent dynamical complexity.

### 3.5 Frequency-dependent chaos threshold erosion

Bifurcation diagrams at representative stimulation frequencies revealed systematic erosion of chaos thresholds with increasing stimulation frequency (Figure 5). Figures 5A–E show bifurcation diagrams at 2, 5, 10, 15, and 50 Hz, respectively. At 2 Hz stimulation, chaotic regions (identified by positive Lyapunov exponents, pink shading) occupied 18 distinct parameter ranges across the β_NMDA_ sweep, with broad ISI distributions spanning multiple oscillatory bands. At 5 Hz (Figure 5B), chaotic windows showed earlier destabilization with ISI compression. At intermediate frequencies (10–15 Hz) (Figures 5C, D), chaotic windows progressively narrowed and shifted toward lower β_NMDA_ values, indicating that faster synaptic drive raises the threshold for chaos onset. At 50 Hz stimulation (Figure 5E), chaos was nearly eliminated entirely, with tight ISI clustering around 15–18 ms corresponding to pure gamma-band firing. Across the analyzed β_NMDA_ range, chaotic windows progressively narrowed and were displaced toward lower β_NMDA_ values with increasing stimulation frequency, substantially reducing the fraction of parameter space susceptible to chaotic dynamics. This frequency-dependent threshold erosion establishes that high-frequency synaptic drive stabilizes neuronal dynamics by constraining the system to periodic orbits, effectively suppressing the period-doubling cascades that generate chaos at lower driving frequencies.

### 3.6 Oscillatory band evolution and spectral consolidation

Frequency band analysis revealed systematic spectral evolution across stimulation rates (Figure 7). Each column (Figures 7A–E) presents an empirical ISI distribution (top) and corresponding Gaussian mixture model fit (bottom) for a single stimulation frequency. ISI distributions were decomposed into physiologically relevant frequency bands: delta (0.5–4 Hz), theta (4–8 Hz), alpha (8–13 Hz), beta (13–30 Hz), and gamma (30–100 Hz). At 2 Hz stimulation (Figure 7A), ISI distributions showed broad multi-band characteristics spanning all five oscillatory categories, with substantial representation in the delta and theta bands reflecting the slow, irregular firing patterns characteristic of chaotic dynamics. As stimulation frequency increased, systematic spectral consolidation occurred: theta and delta components diminished progressively while gamma-band representation expanded. At 10 Hz stimulation (Figure 7C), beta and gamma bands dominated the ISI distribution, with residual alpha and theta contributions. At 15 Hz (Figure 7D), gamma-band firing accounted for the majority of ISIs, with only minor beta-band contributions remaining. At 50 Hz stimulation (Figure 7E), complete gamma band concentration occurred with elimination of all slower frequency components, demonstrating frequency-dependent spectral channeling of neuronal output into a single oscillatory mode. This spectral consolidation has direct functional implications: the transition from multi-band to pure gamma firing represents a shift from a regime capable of encoding information across multiple temporal scales to one dominated by a single, fast oscillatory mode.

### 3.7 GABAergic inhibition provides frequency-selective stabilization

Background GABAergic inhibition systematically modulated neural bifurcation dynamics (Figure 8). Figures 8A–G present the full GABA-frequency bifurcation series at 0, 2, 5, 10, 15, 20, and 50 Hz, respectively, to visualize the progressive stabilization trend at finer resolution. Under control conditions (Figure 8A, 0 Hz GABA), the bifurcation structure exhibited multiple chaotic windows with period-doubling cascades, consistent with the results described in Section 3.3. At low GABA frequencies (Figures 8B, C, 2–5 Hz), minimal stabilization occurred, with chaotic parameter coverage remaining broadly similar to control. Progressive GABA frequency increases demonstrated systematic stabilization: at 10–15 Hz (Figures 8D, E), several chaotic windows were suppressed and the remaining chaotic regions narrowed substantially, indicating that moderate inhibitory tone partially constrains the system's dynamical repertoire. At 20 Hz GABA (Figure 8F), chaotic regions were substantially reduced with ISI ranges constrained to 20–80 ms. At 50 Hz GABA (Figure 8G), complete chaos elimination was achieved across the entire β_NMDA_ range, with all parameter combinations producing regular periodic firing. Quantitatively, GABAergic inhibition expanded stable parameter space by 34.2% while selectively preserving beneficial gamma-band oscillations and suppressing pathological lower-frequency irregularities. This frequency-selective stabilization suggests that GABAergic interneurons serve as natural chaos suppressors in cortical circuits, maintaining firing regularity without eliminating the fast oscillatory dynamics required for normal information processing.

### 3.8 GABA modulates CaMKII-mediated plasticity states

Figure 9 shows that GABAergic inhibition systematically shifts CaMKII-dependent plasticity toward lower phosphorylation states. Final phospho-CaMKII levels were classified into LTD, transition, and LTP regimes, revealing a progressive reduction in high-phosphorylation trajectories as GABA frequency increased (Figures 9A–C). The β_NMDA_ phase diagrams and fixed-GABA sweeps (Figures 9D, E) show contraction of the LTP-permissive region and expansion of LTD-dominated parameter space under inhibitory drive.

This shift is quantified in panel F: under 0 Hz GABA, the parameter space comprised 65% LTD, 25% transition, and 10% LTP; at 20 Hz GABA, this changed to 82% LTD, 15% transition, and 3% LTP; at 50 Hz GABA, LTP was fully eliminated, yielding 82% LTD and 18% transition (2 plasticity regions). These results indicate that GABAergic inhibition constrains NMDA/CaMKII-driven plasticity and preferentially stabilizes depression-like states.

### 3.9 Differential excitatory and inhibitory neuron responses

To examine how NMDA kinetics differentially affect excitatory and inhibitory neurons, we compared pyramidal (excitatory) and fast-spiking (inhibitory) neuron responses across three synaptic input conditions: glutamate only, GABA only, and combined glutamate + GABA (Supplementary Figures S4–S10).

Under glutamate-only stimulation (Supplementary Figures S5A, B), the excitatory neuron fired at 50 Hz with large AMPA- and NMDA-mediated currents. Pure GABAergic input alone was insufficient to generate action potentials in either neuron type (Supplementary Figures S5C, D, S6B), producing only subthreshold hyperpolarization. Combined glutamate + GABA input reduced firing to 40 Hz in the excitatory neuron while the inhibitory neuron maintained 50 Hz (Supplementary Figures S5E, F, S6C), demonstrating differential sensitivity to inhibitory modulation.

Side-by-side comparison under combined input (Supplementary Figure S7) revealed that despite differing firing rates (excitatory: 40 Hz, inhibitory: 50 Hz), the excitatory neuron exhibited larger calcium transients (peak ~0.9 vs. ~0.65 a.u.) and ~4-fold higher CaMKII phosphorylation (~3 × 10^−5^ vs. ~7 × 10^−6^), reflecting stronger NMDA-mediated calcium influx in pyramidal neurons.

Systematic variation of β_NMDA_ (Supplementary Figure S8) showed that both neuron types exhibit monotonically decreasing firing rate and CaMKII phosphorylation with increasing deactivation rate, but the excitatory neuron consistently maintained higher CaMKII levels across the entire β_NMDA_ range. The excitation/inhibition current ratio decreased from ~8 at low β_NMDA_ to ~3 at high β_NMDA_, remaining above the balanced state (E/I = 1) throughout.

Conductance tuning analysis (Supplementary Figure S9) demonstrated that progressive scaling of GABAergic conductance reduced firing rate in both neuron types, with the inhibitory neuron maintaining higher rates at elevated GABA levels. Glutamatergic scaling increased firing with steeper gain in inhibitory neurons, reaching 140 Hz at 4 × baseline. The two-dimensional excitation–inhibition balance heatmaps (Supplementary Figures S9C, D) revealed competitive gradients between excitatory and inhibitory drive, with the inhibitory neuron exhibiting a broader responsive region.

The three-way interaction analysis (β_NMDA_× neuron type × input condition; Supplementary Figure S10) showed that CaMKII phosphorylation differences between excitatory and inhibitory neurons persisted even when firing rates converged under combined input, indicating that the plasticity-relevant calcium–CaMKII pathway remains differentially activated independently of output firing rate.

## 4 Discussion

Our computational analysis reveals that NMDA receptor kinetics fundamentally control neuronal dynamics through dual pathways, with broad implications across multiple neuropsychiatric conditions (Hansen et al., 2021; Paoletti et al., 2013). While these findings have potential applications to schizophrenia, autism spectrum disorders, Alzheimer's disease, and chronic pain syndromes (Gulchina et al., 2017; Zhou and Sheng, 2013; Bruining et al., 2020), we focus our detailed discussion on two specific domains where NMDA dysfunction plays particularly well-characterized roles: addiction-related memory formation and visual processing disorders.

### 4.1 Dual pathways framework

Two mechanistically distinct routes to firing irregularity emerged from our analysis of over 2.9 million ISI observations. Rapid-deactivation irregularity (Pathway 1) occurs under relatively rapid NMDA deactivation (within the upper portion of the analyzed β_NMDA_ window) combined with specific input-frequency conditions, where deterministic instability can emerge in discrete parameter windows and degrade information transfer (MI = 0.185 bits vs. 0.275 bits optimal)(Durstewitz and Gabriel, 2007; Toyoizumi and Abbott, 2011). Prolonged activation irregularity (Pathway 2) results from slow NMDA deactivation (β_NMDA_ < 0.02 ms^−1^) creating instability even under weak drive, with sustained calcium influx driving aberrant CaMKII phosphorylation (Zhabotinsky, 2000; Abraham, 2008).

The identification of an optimal kinetic window (β_NMDA_ = 0.042 ms^−1^; MI = 0.2753 bits) represents a critical balance point that maximizes spike timing information capacity while avoiding pathological states (de Ruyter van Steveninck et al., 1997; Schreiber et al., 2009). This framework challenges traditional views attributing irregular firing solely to synaptic noise or network imbalance, instead revealing NMDA kinetics as master regulators of cortical excitability (Wang, 1999; Ruggiero et al., 2025).

To clarify how these pathways emerge from the preceding Results, we briefly map each pathway to the relevant figures. Pathway 1 (rapid-deactivation irregularity) is visible in the bifurcation diagrams (Figure 4D) as dense ISI scatter in the upper β_NMDA_ range, confirmed by positive MLE values in the heatmaps (Figures 4A, B) and by the progressive collapse of multi-band ISI distributions to broadband chaos (Figure 7). The erosion of chaos thresholds with increasing stimulation frequency (Figure 5) shows that Pathway 1 chaotic windows are amplified under specific drive conditions. Pathway 2 (prolonged activation irregularity) corresponds to the low-β_NMDA_, low-frequency region of the heatmaps where elevated entropy persists even under sparse input (Figures 3, 4A), and is mechanistically linked to sustained Ca^2+^ influx and elevated CaMKII phosphorylation (Figure 9). The optimal kinetic window separating these pathways is identified by the MI peak at β_NMDA_ = 0.042 ms^−1^ (Figure 3), where spike patterns are maximally informative about the kinetic state. GABAergic inhibition preferentially suppresses Pathway 1 chaotic windows while preserving the optimal window (Figures 8, 9). The entropy and mutual information profiles delineate three functionally distinct NMDA kinetic regimes that can be mapped onto healthy and pathological conditions. In the optimal regime (β_NMDA_≈0.042 ms^−1^), moderate entropy coexists with maximal MI, indicating that spike patterns are sufficiently variable to encode information yet structured enough to transmit it reliably—consistent with normal cortical processing. In Pathway 1 (rapid-deactivation regime), entropy remains elevated but MI drops sharply, reflecting a regime where rapid NMDA deactivation produces deterministic chaos that disrupts reliable signal transmission; pharmacologically, this regime may correspond to conditions of NMDA hypofunction such as those implicated in schizophrenia-spectrum disorders. At the upper end of the global β_NMDA_ scan, entropy approaches saturation (Figure 3C), marking the boundary of ISI variability beyond which further increases in deactivation rate cannot increase firing irregularity. In Pathway 2 (β_NMDA_ < 0.02 ms^−1^), prolonged receptor activation sustains calcium influx and aberrant CaMKII phosphorylation, paralleling the NMDA hyperfunction associated with chronic drug exposure and pathological memory consolidation. We focused the systematic parameter sweep on β_NMDA_ rather than β_AMPA_ for several reasons. First, NMDA receptors possess uniquely slow deactivation kinetics (τ_decay_~50–300 ms) compared to AMPA receptors (τ_decay_~1–2 ms), making NMDA kinetics the dominant controller of sustained calcium influx and downstream plasticity cascades. Second, NMDA current is subject to voltage-dependent Mg^2+^ block, creating a nonlinear interaction between membrane dynamics and synaptic conductance that is absent for AMPA receptors. Third, a comprehensive β_AMPA_ sensitivity analysis (Supplementary Figure S3) confirmed that varying AMPA closing rates produces smooth, monotonic changes in firing rate and CaMKII phosphorylation without the period-doubling cascades or chaotic windows observed for β_NMDA_. The two-dimensional β_AMPA_×β_NMDA_ interaction heatmaps (Supplementary Figures S3E, F) further demonstrate that β_NMDA_ is the dominant controller of dynamical complexity, while β_AMPA_ modulates overall excitability level.

### 4.2 Implications for addiction neuroscience

#### 4.2.1 Pathological memory formation

The slow NMDA deactivation pathway (Pathway 2) directly parallels kinetic alterations reported after chronic drug exposure (Kalivas, 2009; Wolf, 2016; Volkow et al., 2016). Our findings indicate that prolonged receptor activation sustains CaMKII phosphorylation at markedly elevated levels compared with the normal-kinetics regime, thereby creating conditions for pathological LTP that differs qualitatively from normal learning-related plasticity (Lüscher and Malenka, 2011; Pascoli et al., 2014).

This prolonged activation enables formation of abnormally persistent drug-associated memories through sustained calcium influx and aberrant plasticity mechanisms (Kauer and Malenka, 2007; Hearing et al., 2016). Unlike normal memory formation requiring precisely timed calcium transients, drug-induced memories exploit aberrantly extended NMDA activation to create difficult-to-reverse synaptic modifications (Borjkhani et al., 2018a,b). The entropy characteristics in this regime suggest this pathway not only creates pathological plasticity but also disrupts normal information processing, potentially explaining cognitive inflexibility observed in addiction (Goldstein and Volkow, 2011). These findings complement our previous work showing that opioids can induce pathological theta oscillations associated with addiction memory formation (Borjkhani et al., 2018c), and extend our understanding of how different drugs of abuse alter neural computation through distinct ionic mechanisms (Borjkhani et al., 2022).

#### 4.2.2 Therapeutic targeting strategies

Our findings suggest novel therapeutic approaches targeting NMDA kinetics during memory reconsolidation (Nader et al., 2000; Lee et al., 2006). The rapid-deactivation chaotic dynamics (Pathway 1) indicate that pharmacologically accelerating receptor deactivation during memory retrieval could disrupt reconsolidation by degrading the neural code required for memory restabilization (Lee et al., 2017). This approach could selectively target drug memories while preserving normal memory function.

The frequency-dependent stability effects have important implications for cue-induced relapse. The systematic frequency-dependent narrowing of chaotic parameter windows suggests that intense cue exposure could destabilize cortical processing and facilitate access to latent drug memories (Buzsáki and Wang, 2012; Fries, 2009). Understanding these thresholds could inform timing and intensity of therapeutic interventions during addiction treatment.

### 4.3 Applications to visual processing disorders

#### 4.3.1 Retinal pathophysiology

NMDA receptors in retinal ganglion cells are critical for contrast sensitivity, direction selectivity, and light adaptation (Manookin et al., 2010; Chen et al., 2016). Our dual-pathway framework reveals mechanistically distinct routes to retinal dysfunction. The prolonged activation pathway maintains sustained calcium influx directly relevant to excitotoxic mechanisms in glaucoma, where chronic glutamate elevation leads to retinal ganglion cell death (Seki and Lipton, 2008; Boccuni and Fairless, 2022). From a retinal degeneration perspective, degenerating retinas exhibit spontaneous network oscillations and correlated retinal ganglion cell spiking that create structured noise capable of distorting visual signaling (Menzler and Zeck, 2011; Margolis et al., 2014). These pathological oscillations, which arise from altered excitatory–inhibitory balance in the deafferented inner retina (Trenholm and Awatramani, 2015), share qualitative features with the oscillatory regimes produced in our model when NMDA kinetics are shifted away from the optimal window. The frequency-dependent transitions we observe (Figure 6)—from irregular multi-band firing at low stimulation frequencies to rhythmic gamma-range activity at high frequencies—are thus directly relevant to understanding how retinal degeneration alters ganglion cell temporal coding and how stimulation-based vision restoration strategies may interact with residual network dynamics.

#### 4.3.2 Cortical visual processing

The systematic oscillatory pattern shifts have direct relevance for cortical visual processing, where gamma oscillations mediate feature binding and attention while alpha rhythms control spatial attention and predictive coding (Jensen and Mazaheri, 2010; Singer, 1999). Our frequency-dependent analysis reveals that NMDA kinetics fundamentally control the balance between these computational modes (Fox and Daw, 1992; Nowak et al., 1997). In our framework, gamma-range signatures emerge when high-frequency stimulation produces short ISIs and rhythmic temporal structure (Figure 7), consistent with narrowband gamma oscillations that propagate and synchronize throughout the thalamocortical visual system (Shin et al., 2023). The transition from multi-band firing at low stimulation frequencies to pure gamma-band concentration at high frequencies (Section 3.6) mirrors the frequency-dependent sharpening of temporal coding observed in visual cortical neurons responding to increasing contrast or temporal frequency (Nowak et al., 1997). Thus, stimulation frequency in our model should be interpreted not merely as “stronger drive” but as a control parameter that selects dynamical regimes with distinct reliability and oscillatory structure relevant to downstream visual information processing.

#### 4.3.3 Correspondence with *in vivo* firing patterns

The qualitative firing regimes produced by our model across the β_NMDA_× frequency parameter space—regular periodic spiking, irregular/unstable firing, and high-frequency gamma-range rhythmicity—are consistent with the range of spiking variability and oscillation-associated temporal patterns observed in large-scale *in vivo* recordings from the mouse visual system Siegle et al., (2021). In particular, (Siegle et al. 2021) reported that units across the visual hierarchy (LGN, V1, higher visual areas) exhibit a broad continuum of firing regularity, with coefficient of variation (CV) of ISI distributions spanning values from near-periodic (CV < 0.5) to highly irregular (CV>1.5), encompassing the range of regimes produced by our β_NMDA_ sweep. Furthermore, the frequency-dependent stabilization we observe—where increasing stimulation frequency progressively constrains firing to gamma-band periodicity—is qualitatively consistent with the narrower ISI distributions and enhanced temporal precision reported for visually driven responses compared to spontaneous activity in cortical recordings (Siegle et al., 2021; Shin et al., 2023). While a full quantitative fitting of model parameters to specific *in vivo* units is beyond the scope of this study and constitutes an important future direction, the qualitative correspondence between our simulated regimes and experimentally observed firing patterns supports the physiological relevance of the β_NMDA_× frequency regime map. To make this comparison explicit and quantitative, Supplementary Table S8 compiles side-by-side ranges of (i) passive membrane properties, (ii) firing-rate and ISI statistics, (iii) intracellular calcium dynamics, (iv) synaptic kinetic time constants, and (v) oscillatory-band characteristics obtained from our simulations vs. values reported from *in vivo* and *in vitro* recordings in cortical pyramidal neurons and the mouse visual system. Across all five categories, the simulated ranges overlap the experimentally reported ranges, supporting the physiological plausibility of the regimes generated across the β_NMDA_×*f*_stim_ parameter space. We emphasize that this is a qualitative plausibility check rather than a formal parameter-estimation procedure, which—as noted in Section 4.7—remains an important future direction.

### 4.4 GABAergic modulation and circuit stabilization

GABAergic inhibition provided frequency-selective stabilization, expanding stable parameter space by 34.2% while producing minimal effects on beneficial gamma rhythms but significant modulation of beta/alpha oscillations (Isaacson and Scanziani, 2011; Carlén et al., 2012). This selective preservation of fast rhythms while dampening pathological slower oscillations suggests that precisely targeted GABAergic interventions could provide therapeutic benefits without compromising normal cognitive function (Gonzalez-Burgos and Lewis, 2008; Lewis et al., 2005).

### 4.5 Clinical translation and precision medicine

The parameter-dependent effects we identify suggest that therapeutic interventions should be tailored to individual NMDA dysfunction patterns rather than employing broad-spectrum approaches (Glasgow et al., 2015; Traynelis et al., 2010).

### 4.6 Comparison with experimental observations

Although experimental studies rarely report an explicit “β_NMDA_” parameter, several observations directly support the mechanisms captured by our β_NMDA_ sweep. First, NMDA receptor conductance and the temporal structure of synaptic input modulate postsynaptic firing variability in cortical neurons; Harsch and Robinson (2000) reported that the NMDA component of compound synaptic events can alter spike-time variability and reliability depending on input synchronization, consistent with our finding that prolonging NMDA activation (smaller β_NMDA_) broadens temporal integration and alters spike-timing regularity. Second, the physiological plausibility of varying NMDA kinetics is supported by measurements of Mg^2+^ unblock dynamics showing fast and slow components that effectively regulate the NMDA contribution to spike generation and temporal integration (Vargas-Caballero and Robinson, 2003, 2004; Kampa et al., 2004). Third, the strong dependence of spike timing on input frequency in visual cortical neurons (Nowak et al., 1997) aligns with our finding that stimulation frequency interacts with NMDA decay kinetics to shift the system between dynamical regimes (cf. Figures 6, 5). Together, these studies provide experimental grounding for interpreting β_NMDA_ as a kinetic control of the NMDA integration window and for comparing our β_NMDA_× frequency regime map to cortical response variability and frequency-dependent processing.

### 4.7 Model limitations and future directions

Several limitations should be acknowledged when interpreting our findings. Our single-cell approach cannot capture network-level phenomena like synchronization and large-scale oscillations crucial for addiction circuits and visual processing networks (Cabral et al., 2014; Anticevic et al., 2012). NMDA receptor subtypes and kinetic properties vary across brain regions, requiring region-specific parameter adjustments for clinical applications (Monyer et al., 1992; Standaert et al., 1999; Sheng et al., 1994). Although we have established qualitative correspondence between our simulated firing regimes and experimentally observed spiking patterns in the visual system (Section 4.3.3), direct quantitative validation—fitting model parameters to specific *in vivo* spike trains and comparing ISI statistics, autocorrelograms, and spectral features on a unit-by-unit basis—remains an important future direction. Such validation, potentially using large-scale datasets such as the Allen Brain Observatory Neuropixels Visual Coding resource (Siegle et al., 2021), would strengthen the translational applicability of the β_NMDA_× frequency regime map and enable patient- or region-specific parameter estimation. An important future extension is the application of nonlinear dimensionality-reduction methods (e.g., UMAP or t-SNE) to the large-scale ISI dataset (over 2.9 million observations) to visualize latent structure and identify clusters associated with specific dynamical regimes and parameter combinations. Such analysis could map emergent clusters to β_NMDA_, stimulation frequency, GABAergic condition, and chaos labels (e.g., positive Lyapunov exponent windows). However, performing this rigorously requires additional methodological development beyond the scope of the present study, including feature design (raw ISIs vs. sequence-level descriptors), balanced sub-sampling or stratification across the parameter space, hyperparameter sensitivity analysis for the embedding algorithm, and quantitative cluster-validation procedures. Although the current combination of entropy, Lyapunov exponent, bifurcation structure, and frequency-band decomposition already provides an interpretable regime-based organization of spike patterns across the β_NMDA_—frequency parameter space, we reserve a dedicated dimensionality-reduction analysis as a follow-up study building on the current mechanistic framework.

## 5 Conclusion

Our computational investigation reveals NMDA receptor kinetics as fundamental controllers of neuronal excitability and synaptic plasticity through dual mechanistic pathways. Analysis of over 2.9 million inter-spike intervals identified two distinct routes to firing irregularity: rapid-deactivation irregularity under fast NMDA kinetics and prolonged-activation irregularity under slow deactivation, with an optimal kinetic window at β_NMDA_ = 0.042 ms^−1^ maximizing information encoding.

These findings provide mechanistic insights into addiction-related memory formation, where prolonged NMDA activation creates conditions for pathological plasticity, and visual processing disorders, where altered kinetics disrupt retinal function and cortical oscillatory balance. GABAergic inhibition offers frequency-selective stabilization, expanding stable parameter space by 34.2% while preserving beneficial gamma rhythms.

## Data Availability

The datasets presented in this study can be found in online repositories. The names of the repository/repositories and accession number(s) can be found below: https://github.com/borjkhani/Bifurcation_NMDA_FCN.

## References

[B1] Abraham W. C. (2008). Metaplasticity: tuning synapses and networks for plasticity. Nat. Rev. Neurosci. 9, 387–399. doi: 10.1038/nrn2356 18401345

[B2] Anticevic A. Gancsos M. Murray J. D. Repovs G. Driesen N. R. Ennis D. J. . (2012). NMDA receptor function in large-scale anticorrelated neural systems with implications for cognition and schizophrenia. Proc. Nat. Acad. Sci. 109, 16720–16725. doi: 10.1073/pnas.1208494109 23012427 PMC3478611

[B3] Bai N. Aida T. Yanagisawa M. Katou S. Sakimura K. Mishina M. . (2013). NMDA receptor subunits have different roles in NMDA-induced neurotoxicity in the retina. Mol. Brain 6:34. doi: 10.1186/1756-6606-6-34 23902942 PMC3733768

[B4] Boccuni I. Fairless R. (2022). Retinal glutamate neurotransmission: from physiology to pathophysiological mechanisms of retinal ganglion cell degeneration. Life 12:638. doi: 10.3390/life12050638 35629305 PMC9147752

[B5] Borjkhani H. Borjkhani M. Sharif M. A. (2022). Investigating the cocaine-induced reduction of potassium current on the generation of action potentials using a computational model. Basic Clin. Neurosci. 13, 15–24. doi: 10.32598/bcn.2021.1150.2 36589017 PMC9790104

[B6] Borjkhani M. Bahrami F. Janahmadi M. (2018a). Assessing the effects of opioids on pathological memory by a computational model. Basic Clin. Neurosci. 9, 275–288. doi: 10.32598/bcn.9.4.275 30519386 PMC6276537

[B7] Borjkhani M. Bahrami F. Janahmadi M. (2018b). Computational modeling of opioid-induced synaptic plasticity in hippocampus. PLoS ONE 13:e0193410. doi: 10.1371/journal.pone.0193410 29513763 PMC5841814

[B8] Borjkhani M. Bahrami F. Janahmadi M. (2018c). Formation of opioid-induced memory and its prevention: a computational study. Front. Comput. Neurosci. 12:63. doi: 10.3389/fncom.2018.00063 30116187 PMC6082946

[B9] Borst A. Theunissen F. E. (1999). Information theory and neural coding. Nat. Neurosci. 2, 947–957. doi: 10.1038/14731 10526332

[B10] Bruining H. Hardstone R. Juarez-Martinez E. L. Sprengers J. Avramiea A.-E. Simpraga S. . (2020). Measurement of excitation-inhibition ratio in autism spectrum disorder using critical brain dynamics. Sci. Rep. 10:9195. doi: 10.1038/s41598-020-65500-4 32513931 PMC7280527

[B11] Buzsáki G. Wang X.-J. (2012). Mechanisms of gamma oscillations. Annu. Rev. Neurosci. 35, 203–225. doi: 10.1146/annurev-neuro-062111-150444 22443509 PMC4049541

[B12] Cabral H. O. Vinck M. Fouquet C. Pennartz C. M. Rondi-Reig L. Battaglia F. P. (2014). Oscillatory dynamics and place field maps reflect hippocampal ensemble processing of sequence and place memory under NMDA receptor control. Neuron 81, 402–415. doi: 10.1016/j.neuron.2013.11.010 24462101

[B13] Carlén M. Meletis K. Siegle J. H. Cardin J. A. Futai K. Vierling-Claassen D. . (2012). A critical role for NMDA receptors in parvalbumin interneurons for gamma rhythm induction and behavior. Mol. Psychiatry 17, 537–548. doi: 10.1038/mp.2011.31 21468034 PMC3335079

[B14] Chen Q. Pei Z. Koren D. Wei W. (2016). Stimulus-dependent recruitment of lateral inhibition underlies retinal direction selectivity. Elife 5:e21053. doi: 10.7554/eLife.21053 27929372 PMC5176353

[B15] Coyle J. T. (2012). NMDA receptor and schizophrenia: a brief history. Schizophr. Bull. 38, 920–926. doi: 10.1093/schbul/sbs076 22987850 PMC3446237

[B16] Das R. K. Freeman T. P. Kamboj S. K. (2013). The effects of N-methyl d-aspartate and β-adrenergic receptor antagonists on the reconsolidation of reward memory: a meta-analysis. Neurosci. Biobehav. Rev. 37, 240–255. doi: 10.1016/j.neubiorev.2012.11.018 23261501

[B17] de Ruyter van Steveninck R. R. Lewen G. D. Strong S. P. Koberle R. Bialek W. (1997). Reproducibility and variability in neural spike trains. Science 275, 1805–1808. doi: 10.1126/science.275.5307.1805 9065407

[B18] Durstewitz D. Gabriel T. (2007). Dynamical basis of irregular spiking in NMDA-driven prefrontal cortex neurons. Cerebral Cortex 17, 894–908. doi: 10.1093/cercor/bhk044 16740581

[B19] Ermentrout G. B. Galán R. F. Urban N. N. (2008). Reliability, synchrony and noise. Trends Neurosci. 31, 428–434. doi: 10.1016/j.tins.2008.06.002 18603311 PMC2574942

[B20] Foss-Feig J. H. Tadin D. Schauder K. B. Cascio C. J. (2013). A substantial and unexpected enhancement of motion perception in autism. J. Neurosci. 33, 8243–8249. doi: 10.1523/JNEUROSCI.1608-12.2013 23658163 PMC3726259

[B21] Fox K. Daw N. (1992). A model for the action of NMDA conductances in the visual cortex. Neural Comput. 4, 59–83. doi: 10.1162/neco.1992.4.1.59

[B22] Fries P. (2009). Neuronal gamma-band synchronization as a fundamental process in cortical computation. Annu. Rev. Neurosci. 32, 209–224. doi: 10.1146/annurev.neuro.051508.135603 19400723

[B23] Gandal M. J. Edgar J. C. Klook K. Siegel S. J. (2012). Gamma synchrony: towards a translational biomarker for the treatment-resistant symptoms of schizophrenia. Neuropharmacology 62, 1504–1518. doi: 10.1016/j.neuropharm.2011.02.007 21349276 PMC3264822

[B24] Glasgow N. G. Siegler Retchless B. Johnson J. W. (2015). Molecular bases of NMDA receptor subtype-dependent properties. J. Physiol. 593, 83–95. doi: 10.1113/jphysiol.2014.273763 25556790 PMC4293056

[B25] Goldstein R. Z. Volkow N. D. (2011). Dysfunction of the prefrontal cortex in addiction: neuroimaging findings and clinical implications. Nat. Rev. Neurosci. 12, 652–669. doi: 10.1038/nrn3119 22011681 PMC3462342

[B26] Golomb D. Yue C. Yaari Y. (2006). Contribution of persistent NA+ current and m-type k+ current to somatic bursting in ca1 pyramidal cells: combined experimental and modeling study. J. Neurophysiol. 96, 1912–1926. doi: 10.1152/jn.00205.2006 16807352

[B27] Gonzalez-Burgos G. Lewis D. A. (2008). GABA neurons and the mechanisms of network oscillations: implications for understanding cortical dysfunction in schizophrenia. Schizophr. Bull. 34, 944–961. doi: 10.1093/schbul/sbn070 18586694 PMC2518635

[B28] Gulchina Y. Xu S.-J. Snyder M. A. Elefant F. Gao W.-J. (2017). Epigenetic mechanisms underlying NMDA receptor hypofunction in the prefrontal cortex of juvenile animals in the MAM model for schizophrenia. J. Neurochem. 143, 320–333. doi: 10.1111/jnc.14101 28628228 PMC5653427

[B29] Hansen K. B. Wollmuth L. P. Bowie D. Furukawa H. Menniti F. S. Sobolevsky A. I. . (2021). Structure, function, and pharmacology of glutamate receptor ion channels. Pharmacol. Rev. 73, 298–487. doi: 10.1124/pharmrev.120.000131 PMC8626789 34753794

[B30] Harsch A. Robinson H. P. (2000). Postsynaptic variability of firing in rat cortical neurons: the roles of input synchronization and synaptic NMDA receptor conductance. J. Neurosci. 20, 6181–6192. doi: 10.1523/JNEUROSCI.20-16-06181.2000 10934268 PMC6772582

[B31] Hearing M. C. Jedynak J. Ebner S. R. Ingebretson A. Asp A. J. Fischer R. A. . (2016). Reversal of morphine-induced cell-type-specific synaptic plasticity in the nucleus accumbens shell blocks reinstatement. Proc. Nat. Acad. Sci. 113, 757–762. doi: 10.1073/pnas.1519248113 26739562 PMC4725472

[B32] Hensch T. K. (2005). Critical period plasticity in local cortical circuits. Nat. Rev. Neurosci. 6, 877–888. doi: 10.1038/nrn1787 16261181

[B33] Hua T. Li X. He L. Zhou Y. Wang Y. Leventhal A. G. (2006). Functional degradation of visual cortical cells in old cats. Neurobiol. Aging 27, 155–162. doi: 10.1016/j.neurobiolaging.2004.11.012 16298251

[B34] Hunt D. L. Castillo P. E. (2012). Synaptic plasticity of NMDA receptors: mechanisms and functional implications. Curr. Opin. Neurobiol. 22, 496–508. doi: 10.1016/j.conb.2012.01.007 22325859 PMC3482462

[B35] Hynd M. R. Scott H. L. Dodd P. R. (2004). Glutamate-mediated excitotoxicity and neurodegeneration in alzheimer's disease. Neurochem. Int. 45, 583–595. doi: 10.1016/j.neuint.2004.03.007 15234100

[B36] Isaacson J. S. Scanziani M. (2011). How inhibition shapes cortical activity. Neuron 72, 231–243. doi: 10.1016/j.neuron.2011.09.027 22017986 PMC3236361

[B37] Jensen O. Mazaheri A. (2010). Shaping functional architecture by oscillatory alpha activity: gating by inhibition. Front. Hum. Neurosci. 4:186. doi: 10.3389/fnhum.2010.00186 21119777 PMC2990626

[B38] Kalivas P. W. (2009). The glutamate homeostasis hypothesis of addiction. Nat. Rev. Neurosci. 10, 561–572. doi: 10.1038/nrn2515 19571793

[B39] Kampa B. M. Clements J. Jonas P. Stuart G. J. (2004). Kinetics of Mg2+ unblock of NMDA receptors: implications for spike-timing dependent synaptic plasticity. J. Physiol. 556, 337–345. doi: 10.1113/jphysiol.2003.058842 14754998 PMC1664940

[B40] Kauer J. A. Malenka R. C. (2007). Synaptic plasticity and addiction. Nat. Rev. Neurosci. 8, 844–858. doi: 10.1038/nrn2234 17948030

[B41] Latremoliere A. Woolf C. J. (2009). Central sensitization: a generator of pain hypersensitivity by central neural plasticity. J. Pain 10, 895–926. doi: 10.1016/j.jpain.2009.06.012 19712899 PMC2750819

[B42] Lee J. L. Milton A. L. Everitt B. J. (2006). Reconsolidation and extinction of conditioned fear: inhibition and potentiation. J. Neurosci. 26, 10051–10056. doi: 10.1523/JNEUROSCI.2466-06.2006 17005868 PMC6674482

[B43] Lee J. L. Nader K. Schiller D. (2017). An update on memory reconsolidation updating. Trends Cogn. Sci. 21, 531–545. doi: 10.1016/j.tics.2017.04.006 28495311 PMC5605913

[B44] Lewis D. A. Hashimoto T. Volk D. W. (2005). Cortical inhibitory neurons and schizophrenia. Nat. Rev. Neurosci. 6, 312–324. doi: 10.1038/nrn1648 15803162

[B45] Lüscher C. Malenka R. C. (2011). Drug-evoked synaptic plasticity in addiction: from molecular changes to circuit remodeling. Neuron 69, 650–663. doi: 10.1016/j.neuron.2011.01.017 21338877 PMC4046255

[B46] Lüscher C. Malenka R. C. (2012). NMDA receptor-dependent long-term potentiation and long-term depression (LTP/LTD). Cold Spring Harb. Perspect. Biol. 4:a005710. doi: 10.1101/cshperspect.a005710 PMC3367554 22510460

[B47] Manookin M. B. Weick M. Stafford B. K. Demb J. B. (2010). NMDA receptor contributions to visual contrast coding. Neuron 67, 280–293. doi: 10.1016/j.neuron.2010.06.020 20670835 PMC2913150

[B48] Margolis D. J. Gartland A. J. Singer J. H. Detwiler P. B. (2014). Network oscillations drive correlated spiking of ON and OFF ganglion cells in the rd1 mouse model of retinal degeneration. PLoS ONE 9:e86253. doi: 10.1371/journal.pone.0086253 24489706 PMC3904909

[B49] Markram H. Lübke J. Frotscher M. Sakmann B. (1997). Regulation of synaptic efficacy by coincidence of postsynaptic aps and epsps. Science 275, 213–215. doi: 10.1126/science.275.5297.213 8985014

[B50] Menzler J. Zeck G. (2011). Network oscillations in rod-degenerated mouse retinas. J. Neurosci. 31, 2280–2291. doi: 10.1523/JNEUROSCI.4238-10.2011 21307264 PMC6633031

[B51] Monyer H. Sprengel R. Schoepfer R. Herb A. Higuchi M. Lomeli H. . (1992). Heteromeric NMDA receptors: molecular and functional distinction of subtypes. Science. 256, 1217–1221. doi: 10.1126/science.256.5060.1217 1350383

[B52] Morishita H. Hensch T. K. (2008). Critical period revisited: impact on vision. Curr. Opin. Neurobiol. 18, 101–107. doi: 10.1016/j.conb.2008.05.009 18534841

[B53] Nader K. Schafe G. E. Le Doux J. E. (2000). Fear memories require protein synthesis in the amygdala for reconsolidation after retrieval. Nature 406, 722–726. doi: 10.1038/35021052 10963596

[B54] Nowak L. G. Sanchez-Vives M. V. McCormick D. A. (1997). Influence of low and high frequency inputs on spike timing in visual cortical neurons. Cerebral Cortex 7, 487–501. doi: 10.1093/cercor/7.6.487 9276174

[B55] Paoletti P. Bellone C. Zhou Q. (2013). NMDA receptor subunit diversity: impact on receptor properties, synaptic plasticity and disease. Nat. Rev. Neurosci. 14, 383–400. doi: 10.1038/nrn3504 23686171

[B56] Pascoli V. Terrier J. Espallergues J. Valjent E. O'Connor E. C. Lüscher C. (2014). Contrasting forms of cocaine-evoked plasticity control components of relapse. Nature 509, 459–464. doi: 10.1038/nature13257 24848058

[B57] Rieke F. Warland D. de Ruyter van Steveninck R. Bialek W. (1997). Spikes: Exploring the Neural Code. Cambridge, MA: MIT Press.

[B58] Robertson C. E. Baron-Cohen S. (2017). Sensory perception in autism. Nat. Rev. Neurosci. 18, 671–684. doi: 10.1038/nrn.2017.112 28951611

[B59] Rubenstein J. L. Merzenich M. M. (2003). Model of autism: increased ratio of excitation/inhibition in key neural systems. Genes, Brain Behav. 2, 255–267. doi: 10.1034/j.1601-183X.2003.00037.x 14606691 PMC6748642

[B60] Ruggiero A. Heim L. R. Susman L. Hreaky D. Shapira I. Katsenelson M. . (2025). NMDA receptors regulate the firing rate set point of hippocampal circuits without altering single-cell dynamics. Neuron 113, 244–259.e7. doi: 10.1016/j.neuron.2024.10.014 39515323

[B61] Santucci D. M. Raghavachari S. (2008). The effects of NR2 subunit-dependent NMDA receptor kinetics on synaptic transmission and CaMKII activation. PLoS Comput. Biol. 4:e1000208. doi: 10.1371/journal.pcbi.1000208 18974824 PMC2563690

[B62] Schreiber S. Samengo I. Herz A. V. (2009). Two distinct mechanisms shape the reliability of neural responses. J. Neurophysiol. 101, 2239–2251. doi: 10.1152/jn.90711.2008 19193775

[B63] Seki M. Lipton S. A. (2008). Targeting excitotoxic/free radical signaling pathways for therapeutic intervention in glaucoma. Prog. Brain Res. 173, 495–510. doi: 10.1016/S0079-6123(08)01134-5 18929130

[B64] Sheng M. Cummings J. Roldan L. A. Jan Y. N. Jan L. Y. (1994). Changing subunit composition of heteromeric NMDA receptors during development of rat cortex. Nature 368, 144–147. doi: 10.1038/368144a0 8139656

[B65] Shin D. Peelman K. Lien A. D. Del Rosario J. Haider B. (2023). Narrowband gamma oscillations propagate and synchronize throughout the mouse thalamocortical visual system. Neuron 111, 1076–1085.e8. doi: 10.1016/j.neuron.2023.03.006 37023711 PMC10112544

[B66] Siegle J. H. Jia X. Durand S. Gale S. Bennett C. Graddis N. . (2021). Survey of spiking in the mouse visual system reveals functional hierarchy. Nature 592, 86–92. doi: 10.1038/s41586-020-03171-x 33473216 PMC10399640

[B67] Singer W. (1999). Neuronal synchrony: a versatile code for the definition of relations? Neuron 24, 49–65. doi: 10.1016/S0896-6273(00)80821-1 10677026

[B68] Snyder E. M. Nong Y. Almeida C. G. Paul S. Moran T. Choi E. Y. . (2005). Regulation of NMDA receptor trafficking by amyloid-β. Nat. Neurosci. 8, 1051–1058. doi: 10.1038/nn1503 16025111

[B69] Soudry D. Meir R. (2012). Conductance-based neuron models and the slow dynamics of excitability. Front. Comput. Neurosci. 6:4. doi: 10.3389/fncom.2012.00004 22355288 PMC3280430

[B70] Standaert D. G. Landwehrmeyer G. B. Kerner J. A. Penney Jr J. B. Young A. B. (1999). Expression of NMDA glutamate receptor subunit mrnas in neurochemically identified projection and interneurons in the striatum of the rat. Molec. Brain Res. 64, 11–23. doi: 10.1016/S0169-328X(98)00293-9 9889300

[B71] Toyoizumi T. Abbott L. F. (2011). Beyond the edge of chaos: amplification and temporal integration by recurrent networks in the chaotic regime. Phys. Rev. E 84:051908. doi: 10.1103/PhysRevE.84.051908 PMC5558624 22181445

[B72] Traynelis S. F. Wollmuth L. P. McBain C. J. Menniti F. S. Vance K. M. Ogden K. K. . (2010). Glutamate receptor ion channels: structure, regulation, and function. Pharmacol. Rev. 62, 405–496. doi: 10.1124/pr.109.002451 20716669 PMC2964903

[B73] Trenholm S. Awatramani G. B. (2015). Origins of spontaneous activity in the degenerating retina. Front. Cell. Neurosci. 9:277. doi: 10.3389/fncel.2015.00277 26283914 PMC4518194

[B74] Vargas-Caballero M. Robinson H. P. (2003). A slow fraction of Mg2+ unblock of NMDA receptors limits their contribution to spike generation in cortical pyramidal neurons. J. Neurophysiol. 89, 2778–2783. doi: 10.1152/jn.01038.2002 12611983

[B75] Vargas-Caballero M. Robinson H. P. (2004). Fast and slow voltage-dependent dynamics of magnesium block in the NMDA receptor: the asymmetric trapping block model. J. Neurosci. 24, 6171–6180. doi: 10.1523/JNEUROSCI.1380-04.2004 15240809 PMC6729657

[B76] Volkow N. D. Koob G. F. McLellan A. T. (2016). Neurobiologic advances from the brain disease model of addiction. N. Engl. J. Med. 374, 363–371. doi: 10.1056/NEJMra1511480 26816013 PMC6135257

[B77] Wang R. Reddy P. H. (2017). Role of glutamate and NMDA receptors in Alzheimer's disease. J. Alzheimer's Dis. 57, 1041–1048. doi: 10.3233/JAD-160763 27662322 PMC5791143

[B78] Wang X.-J. (1999). Synaptic basis of cortical persistent activity: the importance of NMDA receptors to working memory. J. Neurosci. 19, 9587–9603. doi: 10.1523/JNEUROSCI.19-21-09587.1999 10531461 PMC6782911

[B79] Wolf A. Swift J. B. Swinney H. L. Vastano J. A. (1985). Determining lyapunov exponents from a time series. Physica D 16, 285–317. doi: 10.1016/0167-2789(85)90011-9

[B80] Wolf M. E. (2016). Synaptic mechanisms underlying persistent cocaine craving. Nat. Rev. Neurosci. 17, 351–365. doi: 10.1038/nrn.2016.39 27150400 PMC5466704

[B81] Zhabotinsky A. M. (2000). Bistability in the Ca2+/calmodulin-dependent protein kinase-phosphatase system. Biophys. J. 79, 2211–2221. doi: 10.1016/S0006-3495(00)76469-1 11053103 PMC1301111

[B82] Zhou Q. Sheng M. (2013). NMDA receptors in nervous system diseases. Neuropharmacology 74, 69–75. doi: 10.1016/j.neuropharm.2013.03.030 23583930

[B83] Zhuo M. (2016). Neural mechanisms underlying anxiety-chronic pain interactions. Trends Neurosci. 39, 136–145. doi: 10.1016/j.tins.2016.01.006 26878750

