# Supplementary material for: NMDA receptor kinetics drive distinct routes to chaotic firing in pyramidal neurons
Source: Front Comput Neurosci. 2026 Jun 3;20:1753444. doi: 10.3389/fncom.2026.1753444 (PMC13272316; doi:10.3389/fncom.2026.1753444)
Supplement: Supplementary file 1 [file Data_Sheet_1.pdf]

Supplementary Material

for

"NMDA Receptor Kinetics Drive Distinct Routes to Chaotic Firing in Pyramidal Neurons"

Mehdi Borjkhani*, Hadi Borjkhani, Morteza A. Sharif, Fariba Bahrami, Mahyar Janahmadi

Frontiers in Computational Neuroscience

*Correspondence: mborjkhani@ichf.edu.pl

Table S1: **Model state variables and their descriptions.** Complete list of all dynamical variables in the single-compartment pyramidal neuron model. Variables are grouped by functional category: membrane potential, intrinsic ionic gating, synaptic gating, calcium dynamics, and CaMKII phosphorylation.

**Variable**	**Description**	**Units**	**Equation**
Membrane potential			
V	Membrane potential	mV	Eq. (1)
Intrinsic ionic channel gating variables			
m	Na^+ activation (instantaneous)	–	$m = m_\infty(V)$
h	Na^+ inactivation	–	Eq. (5)
p	Persistent Na^+ activation (instantaneous)	–	$p = p_\infty(V)$
n	Delayed rectifier K^+ activation	–	Eq. (9)
a	A-type K^+ activation (instantaneous)	–	$a = a_\infty(V)$
b	A-type K^+ inactivation	–	Eq. (12)
z	M-type K^+ activation	–	Eq. (14)
r	Ca^{2+} channel activation	–	Eq. (16)
c	Fast Ca^{2+}-activated K^+ activation	–	Eq. (18)
d	Fast Ca^{2+}-activated K^+ Ca-dependent (instantaneous)	–	$d = d_\infty([Ca^{2+}]_i)$
q	Slow AHP K^+ activation	–	Eq. (21)
Synaptic receptor gating variables			
m_{AMPA}	AMPA receptor activation	–	Eq. (26)
m_{NMDA}	NMDA receptor activation	–	Eq. (29)
m_{GABA}	$GABA_A$ receptor activation	–	Eq. (31)
$g_{\mathrm{VD,post}}$	NMDA voltage-dependent postsynaptic conductance	–	see code†
Calcium dynamics			
$[Ca^{2+}]_i$	Intracellular calcium concentration	μM	Eq. (24)
CaMKII phosphorylation cascade			
P_0	Unphosphorylated CaMKII fraction	–	Eq. (33)
P_1–P_{10}	i-fold phosphorylated CaMKII fractions	–	Eq. (33)
e_p	Active PP1 concentration	μM	see Methods
I_1	Inhibitor-1 concentration	μM	see Methods

†An additional voltage-dependent postsynaptic NMDA conductance factor: $g'_{\mathrm{VD,post}} = (g_{\mathrm{VD},\infty} - g_{\mathrm{VD,post}})/0.05$, where $g_{\mathrm{VD},\infty} = 0.0007(V + 100)$ and total NMDA conductance is modulated by $(g_{\mathrm{VD,post}} + 1)$.

Table S2: **Intrinsic ionic conductance parameters.** Maximal conductance densities and reversal potentials for all intrinsic ionic currents. Values from Golomb et al. (2006) with modifications as noted.

**Current**	**Symbol**	$\bar{g}$ **(mS/cm²)**	$E_{\mathbf{rev}}$ **(mV)**	**Description**
Transient Na^+	I_{Na}	50	55	Fast spike initiation
Persistent Na^+	I_{NaP}	0.2	55	Subthreshold depolarization
Delayed rectifier K^+	I_{Kdr}	10	−90	Spike repolarization
A-type K^+	I_{A}	1	−90	Transient outward current
M-type K^+	I_{M}	0.5	−90	Slow voltage-dependent K^+
High-voltage Ca^{2+}	I_{Ca}	0.1	120	Ca^{2+} influx
Fast Ca^{2+}-activated K^+	I_{C}	5	−90	Fast afterhyperpolarization
Slow AHP	I_{sAHP}	2	−90	Spike-frequency adaptation
Leak	I_{L}	0.1	−70	Resting conductance

Table S3: **Gating variable kinetic parameters.** Half-activation voltages (θ), slope factors (σ), and time constants (τ) for all voltage-dependent gating variables. Steady-state activation/inactivation follows the Boltzmann form: $x_\infty(V) = [1 + \exp(-(V - \theta_x)/\sigma_x)]^{-1}$.

**Variable**	θ **(mV)**	σ **(mV)**	τ **(ms)**	**Dynamics**	**Current**
m	−35	8	instantaneous	$m = m_\infty(V)$	I_{Na}
h	−50	−6	$\tau_h(V)^\dagger$	differential	I_{Na}
p	−40	2	instantaneous	$p = p_\infty(V)$	I_{NaP}
n	−40	12	$\tau_n(V)^\ddagger$	differential	I_{Kdr}
a	−55	15	instantaneous	$a = a_\infty(V)$	I_{A}
b	−85	−5	15	differential	I_{A}
z	−45	4	75	differential	I_{M}
r	−25	8	1	differential	I_{Ca}
c	−35	5	2	differential	I_{C}
q	–	–	450	$q_\infty([\mathrm{Ca}^{2+}]_i)^\S$	I_{sAHP}
d	–	–	instantaneous	$d_\infty([\mathrm{Ca}^{2+}]_i)^\P$	I_{C}

$^\dagger\tau_h(V) = 0.1 + 0.75 \cdot [1 + \exp(-(V + 45)/(-6))]^{-1}$ ms
$^\ddagger\tau_n(V) = 0.1 + 0.5 \cdot [1 + \exp(-(V + 30)/(-15))]^{-1}$ ms
$^\S q_\infty = [1 + 24/[\mathrm{Ca}^{2+}]_i^4]^{-1}$
$^\P d_\infty = [1 + 6/[\mathrm{Ca}^{2+}]_i]^{-1}$

Table S4: **Synaptic receptor parameters.** Kinetic rate constants, conductances, and reversal potentials for all three receptor types modeled. The NMDA closing rate β_{NMDA} is the primary bifurcation parameter varied systematically across simulations.

Parameter	Symbol	Value	Units
AMPA receptor			
Opening rate	α_{AMPA}	1.1	$\text{mM}^{-1}\text{ms}^{-1}$
Closing rate	β_{AMPA}	0.67	ms^{-1}
Maximal conductance	g_{AMPA}	0.35	nS
Reversal potential	E_{AMPA}	55	mV
Scaling factor	k_{AMPA}	1	–
NMDA receptor			
Opening rate	α_{NMDA}	0.38	$\text{mM}^{-1}\text{ms}^{-1}$
Closing rate	β_{NMDA}	0.002–0.1	ms^{-1}
Scaling factor	k_{NMDA}	1	–
Extracellular [Mg^{2+}]	$[Mg^{2+}]_o$	1.4	mM
Mg^{2+} block constant	–	3.75	mM
Mg^{2+} voltage sensitivity	–	0.062	mV^{-1}
VD postsynaptic gain	–	0.0007	mV^{-1}
VD postsynaptic offset	–	−100	mV
VD time constant	–	0.05	ms
$GABA_A$ receptor			
Opening rate	α_{GABA}	5	$\text{mM}^{-1}\text{ms}^{-1}$
Closing rate	β_{GABA}	0.18	ms^{-1}
Maximal conductance	g_{GABA}	0.35	nS
Reversal potential	E_{GABA}	−80	mV

Table S5: **CaMKII phosphorylation cascade parameters.** All rate constants and concentrations governing the 10-subunit CaMKII phosphorylation model, adapted from Zhabotinsky (2000) and Borjkhani et al. (2018a,b).

Parameter	Symbol	Value	Units
CaMKII phosphorylation rates			
Autophosphorylation rate	k_1	0.5×10^{-3}	s^{-1}
PP1 catalytic rate	k_2	10×10^{-3}	s^{-1}
PP1–I1P association rate	k_3	1×10^{-3}	$\mu\text{M}^{-1}\text{s}^{-1}$
PP1–I1P dissociation rate	k_4	1×10^{-6}	s^{-1}
Ca^{2+} Hill constant (CaMKII)	K_{H1}	4.0	μM
Hill coefficient	n_{H1}	3	–
Michaelis constant (PP1)	K_M	20	μM
Regulatory enzyme parameters			
I1 dephosphorylation rate (calcineurin)	v_{CaN}	2.0×10^{-3}	s^{-1}
Ca^{2+} Hill constant (calcineurin)	K_{H2}	0.7	μM
I1 phosphorylation rate (PKA)	v_{PKA}	0.45×10^{-3}	μM/s
PKA kinetic constant	k_{pk}	0.0059	–
I1-dependent regulation rate	k_I	0.001	–
Initial conditions			
PP1 initial active fraction	$e_p(0)$	1.0	–
I1 initial concentration	$I_1(0)$	0.0	μM
Unphosphorylated CaMKII	$P_0(0)$	1.0	–
Phosphorylated fractions	$P_1(0)$–$P_{10}(0)$	0.0	–

Table S6: **Initial conditions and simulation parameters.** Initial values for all state variables and key numerical integration parameters.

**Variable**	**Symbol**	**Initial Value**	**Units**	**Rationale**
State variable initial conditions				
Membrane potential	$V(0)$	-70	mV	Near resting potential
Na^+ activation	$m(0)$	0.1	–	Near $m_\infty(-70)$
Na^+ inactivation	$h(0)$	0.98	–	Near $h_\infty(-70)$
K^+ DR activation	$n(0)$	0.01	–	Near $n_\infty(-70)$
A-type inactivation	$b(0)$	0.0	–	De-inactivated
M-type activation	$z(0)$	0.05	–	Near $z_\infty(-70)$
AMPA gating	$m_{\mathrm{AMPA}}(0)$	0.0	–	No initial activation
NMDA gating	$m_{\mathrm{NMDA}}(0)$	0.0	–	No initial activation
GABA gating	$m_{\mathrm{GABA}}(0)$	0.0	–	No initial activation
Ca^{2+}	$[\mathrm{Ca}^{2+}]_i(0)$	0.0	μM	Resting baseline
Simulation parameters				
Total duration	$T_{\max}$	10,000	ms	–
Integration timestep	Δt	0.05	ms	RK4 method
Transient discard	–	2,000	ms	Equilibration period
Membrane capacitance	C_m	1.0	μF/cm^2	Standard value
Stimulation parameters				
Glutamate pulse amplitude	G_{glu}	1.0	μM	–
Glutamate pulse width	–	5	ms	Rectangular pulse
Glutamate frequency range	–	1–250	Hz	25 values
GABA pulse amplitude	G_{GABA}	1.0	μM	–
GABA pulse width	–	5	ms	Rectangular pulse
GABA frequency levels	–	0, 2, 5, 10, 15, 20, 50	Hz	7 conditions
Calcium dynamics coefficients				
Ca^{2+} influx via I_{Ca}	–	0.13	μM·cm^2/(mS·ms)	–
Ca^{2+} influx via I_{NMDA}	–	0.012	μM·cm^2/(mS·ms)	–
Ca^{2+} influx via I_{AMPA}	–	0.0012	μM·cm^2/(mS·ms)	–
Ca^{2+} decay time constant	τ_{Ca}	13	ms	Extrusion/buffering

Table S7: **Parameter space exploration summary.** Ranges and resolution for the systematic parameter sweep used in the bifurcation and information-theoretic analyses.

**Parameter**	**Range**	**Steps**	**Spacing**	**N values**
β_{NMDA} (ms^{-1})	0.002–0.1	0.0001	Linear	$\sim$980
Glutamate frequency (Hz)	1–250	–	Logarithmic	25
GABA frequency (Hz)	{0, 2, 5, 10, 15, 20, 50}	–	Discrete	7
Replications per condition	–	–	–	10
Total unique parameter combinations				1,500+
Total simulations (with replications)				15,000+
Total ISI observations after QC				2,942,093

Table S8: **Simulated and experimentally reported values for principal model quantities.** Side-by-side comparison of (A) passive and active membrane properties, (B) firing-rate and ISI statistics, (C) intracellular calcium dynamics, (D) synaptic kinetic time constants, and (E) oscillatory-band characteristics. Simulated values are taken from the baseline conditions and parameter sweeps reported in the main text (principally Figures 3, 4, 5, 7, and 8), evaluated over the focused window $\beta_{\mathrm{NMDA}} \in [0.002, 0.1]$ ms^{-1} and stimulation frequencies 2–50 Hz. Experimental ranges are reported for layer 2/3 and layer 5 cortical pyramidal neurons and, where appropriate, for the mouse visual system at the population level. Ranges rather than point estimates are given.

Quantity	Simulation (this study)	Experimental range	Experimental source	Source in this study
A. Passive and active membrane properties				
Resting membrane potential	-65 ± 5 mV	-65 to -75 mV (L2/3 and L5 pyramidal)	Markram et al. (1997)	Fig. 3C
Action potential amplitude	80–100 mV	70–110 mV (peak-to-peak, somatic)	Markram et al. (1997)	Fig. 3C
Spike threshold	-45 ± 3 mV	-45 to -55 mV	Markram et al. (1997)	Fig. 3C
Adaptation ratio (sustained injection)	0.6–0.8	0.5–0.85 (regular-spiking pyramidal)	Markram et al. (1997)	Sec. 2.1.1
B. Firing-rate and ISI statistics				
Firing rate, representative condition ($\beta_{\mathrm{NMDA}} = 0.052$ ms^{-1}, low-freq. drive)	13.3 Hz	5–40 Hz (visually driven V1 pyramidal)	Siegle et al. (2021)	Fig. 3C
Firing rate, high-frequency drive (50 Hz stim.)	Entrained, ISI $\approx$ 15–18 ms ($\sim$55–65 Hz)	Narrowband gamma firing in V1 at 50–70 Hz	Siegle et al. (2021); Shin et al. (2023)	Figs. 7E, 8E
ISI coefficient of variation (across β_{NMDA} sweep)	$\sim$0.3–1.6 (periodic to chaotic regimes)	0.4–1.8 across visual hierarchy (LGN, V1, higher visual)	Siegle et al. (2021)	Figs. 5D, 7A–E
ISI distribution shape	Unimodal (periodic) to broad / multi-modal (chaotic)	Continuum from near-periodic to highly irregular	Siegle et al. (2021)	Figs. 5E, 8A–E
C. Intracellular calcium dynamics (somatic)				
Resting / mean $[\mathrm{Ca}^{2+}]_i$	0.094 μM (mean, spiking regime)	0.05–0.1 μM (resting, somatic)	Helmchen et al. (1996)	Fig. 3D
Peak $[\mathrm{Ca}^{2+}]_i$ during spiking	0.963 μM	0.3–2 μM (somatic, single to short burst APs)	Helmchen et al. (1996)	Fig. 3D
Calcium transient decay time constant	$\tau_{\mathrm{Ca}} = 13$ ms (Table S6)	100–500 ms (somatic, cortical pyramidal)	Helmchen et al. (1996)	Eq. (24), Table S6
D. Synaptic kinetic time constants				
NMDA effective deactivation, $\tau_{\mathrm{NMDA}} \approx 1/\beta_{\mathrm{NMDA}}$	$\sim$10–500 ms across sweep	50–300 ms (GluN2A/B-containing receptors)	Kampa et al. (2004); Vargas-Caballero and Robinson (2004)	Sec. 2.5.2, Fig. 4

Continued on next page

Table S8 continued from previous page

Quantity	Simulation (this study)	Experimental range	Experimental source	Source in this study
AMPA deactivation time constant, $1/\beta_{\mathrm{AMPA}}$	≈1.5 ms (Table S4)	1–3 ms (fast AMPA EPSC decay)	Kampa et al. (2004)	Table S4
GABA_A deactivation time constant, $1/\beta_{\mathrm{GABA}}$	≈5.6 ms (Table S4)	5–20 ms (fast GABA_A IPSC decay)	Isaacson and Scanziani (2011)	Table S4
E. Oscillatory-band characteristics (ISI-based mapping)				
Gamma band (30–100 Hz, ISI 7–33 ms)	Dominant at stim. freq. ≥15 Hz; ISI clustering 15–18 ms at 50 Hz stim.	Narrowband gamma 50–70 Hz in mouse V1/LGN; broadband gamma 30–90 Hz	Shin et al. (2023); Siegle et al. (2021)	Figs. 7E, 8E
Beta band (13–30 Hz, ISI 33–77 ms)	Emerges at 5–10 Hz stim. in multi-band regime	13–30 Hz cortical beta oscillations	Jensen and Mazaheri (2010)	Fig. 8B–C
Alpha band (8–13 Hz, ISI 77–125 ms)	Subdominant at low-freq. stim.	8–13 Hz cortical alpha rhythm	Jensen and Mazaheri (2010)	Fig. 8A–B
Theta / delta contributions	Present at 2 Hz stim. and low-β_{NMDA}, chaotic regimes	0.5–8 Hz slow oscillations in cortical recordings	Buzsáki and Wang (2012)	Fig. 8A

Supplementary Figures

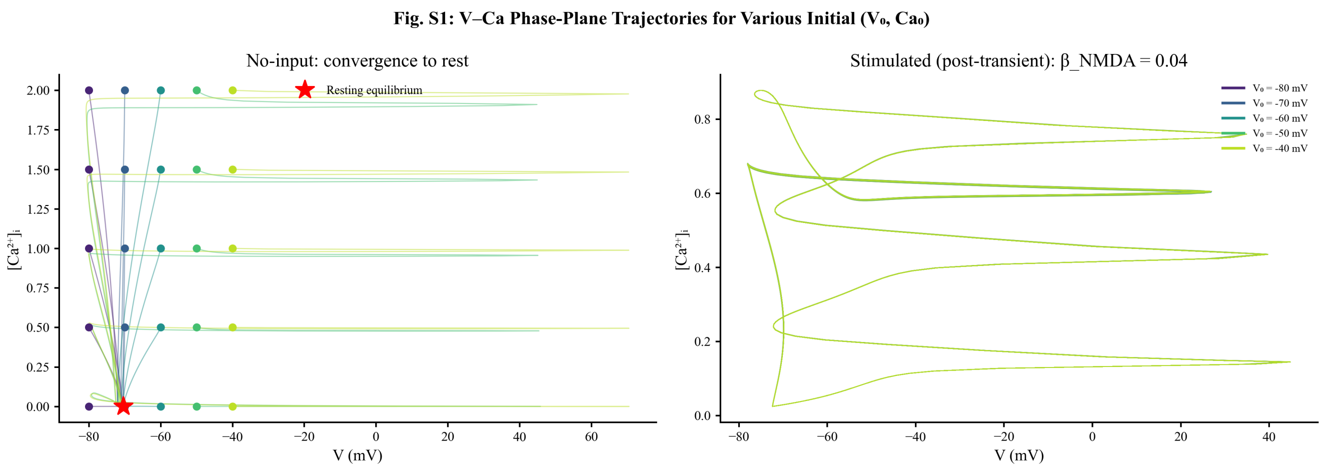

Figure S1: **Sensitivity of model dynamics to initial membrane potential and calcium concentration.** *Left:* V–$[\text{Ca}^{2+}]_i$ phase-plane trajectories under no-input conditions for 25 combinations of initial voltage ($V_0 = -80, -70, -60, -50, -40$ mV) and initial calcium ($[\text{Ca}^{2+}]_{i,0} = 0, 0.5, 1.0, 1.5, 2.0$ μM). All trajectories converge to the same resting equilibrium (red star at approximately $V \approx -75$ mV, $[\text{Ca}^{2+}]_i \approx 0$ μM), demonstrating a unique, globally attracting fixed point. Colored dots indicate initial conditions; line colors encode V_0. *Right:* Post-transient phase-plane trajectories under glutamatergic stimulation at $\beta_{\text{NMDA}} = 0.04$ ms^{-1} for five initial voltages. After the 2000 ms transient discard period, all trajectories converge to the same limit cycle regardless of initial conditions, confirming that the reported bifurcation dynamics and ISI distributions are independent of initial state selection.

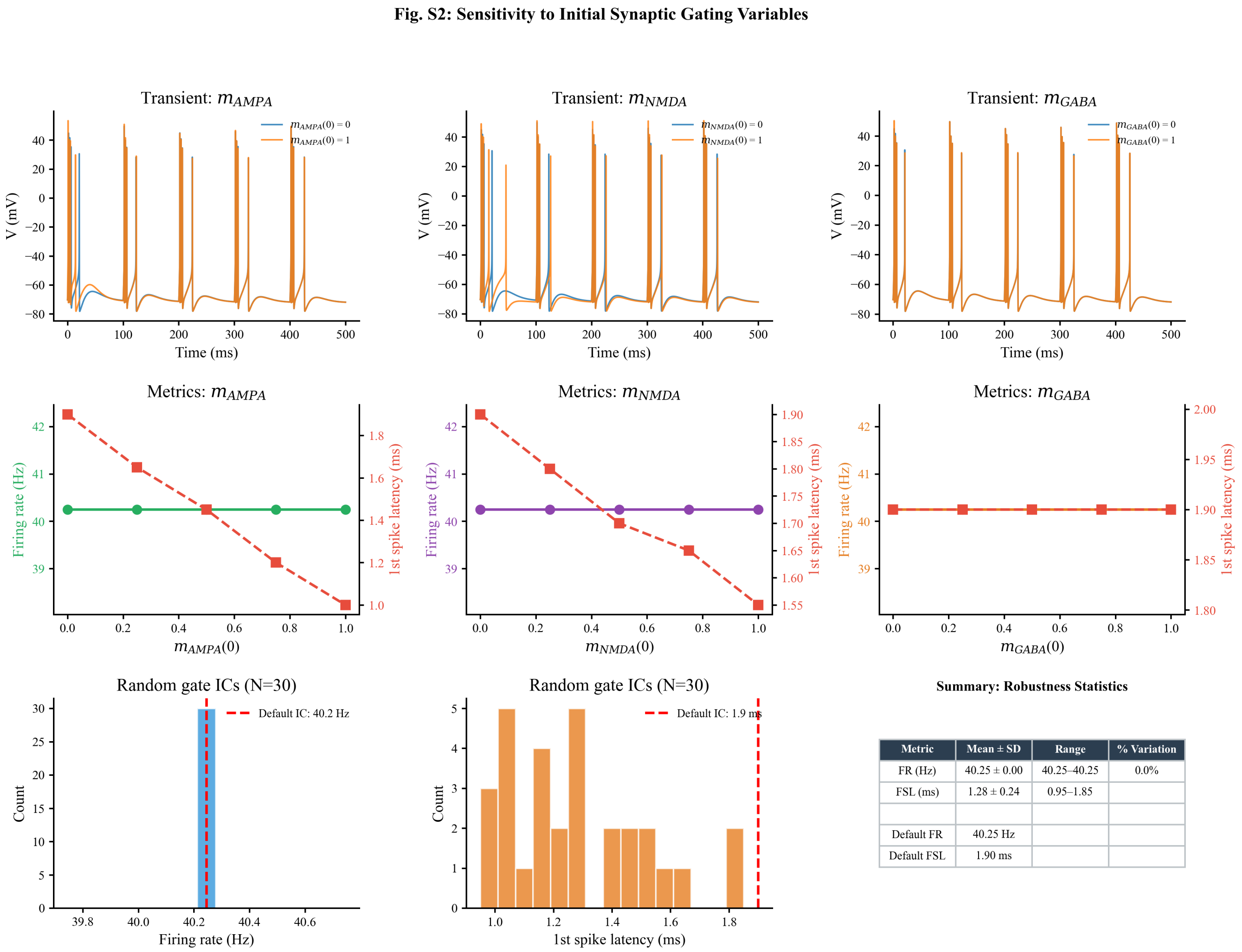

Figure S2: **Sensitivity of model dynamics to initial synaptic gating variables.** *Top row:* Membrane potential traces for extreme initial conditions of $m_{\mathrm{AMPA}}(0)$, $m_{\mathrm{NMDA}}(0)$, and $m_{\mathrm{GABA}}(0)$ (values of 0 and 1). Differences are confined to the first 50–100 ms transient and fully dissipate within the 2000 ms equilibration period. *Middle row:* Steady-state firing rate (green/purple circles) and first spike latency (red squares) as functions of each initial gating variable, systematically varied from 0 to 1. Firing rate is invariant (40.25 Hz for all conditions), while first spike latency shows modest sensitivity to $m_{\mathrm{NMDA}}(0)$ only during the initial transient. *Bottom row:* Distribution of firing rates and first spike latencies across 30 simulations with uniformly random initial gating variables (m_{AMPA}, m_{NMDA}, m_{GABA} each drawn from $U[0, 1]$). Firing rate: 40.25 ± 0.00 Hz (0.0% variation); first spike latency: 1.28 ± 0.24 ms. The summary table confirms that model outputs are robust to initial synaptic gating variable selection.

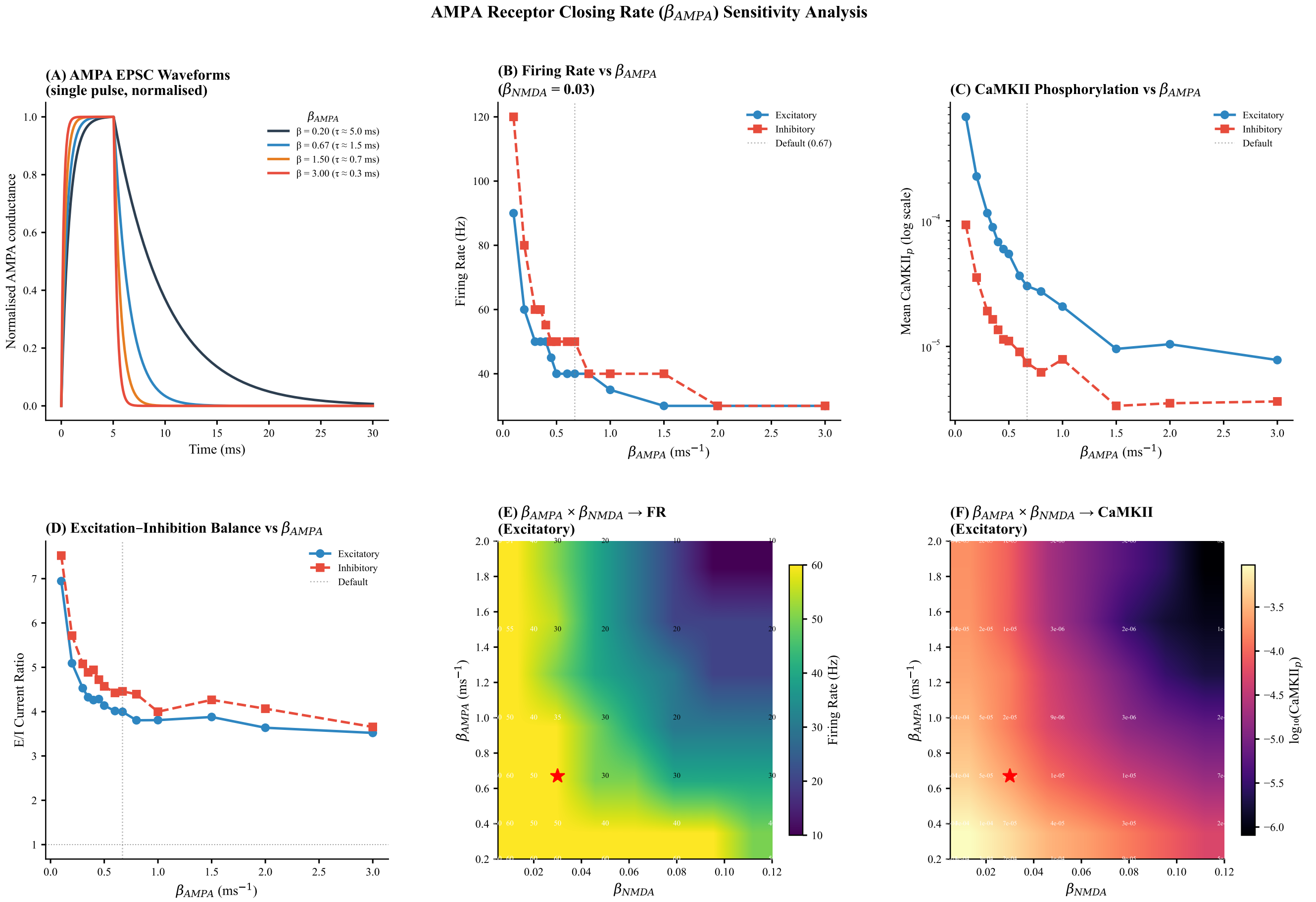

Figure S3: **AMPA receptor closing rate (β_{AMPA}) sensitivity analysis.** (A) Normalized AMPA EPSC waveforms for four representative β_{AMPA} values showing the decay time course. (B) Firing rate as a function of β_{AMPA} at fixed $\beta_{\mathrm{NMDA}} = 0.03$ ms^{-1} for excitatory (pyramidal) and inhibitory (fast-spiking) neurons. Dashed vertical line marks the default value ($\beta_{\mathrm{AMPA}} = 0.67$ ms^{-1}). (C) Mean CaMKII phosphorylation level (log scale) as a function of β_{AMPA}, showing monotonic decrease for both neuron types. (D) Excitation–inhibition current ratio as a function of β_{AMPA}. (E–F) Two-dimensional interaction heatmaps showing the combined effect of β_{AMPA} and β_{NMDA} on firing rate (E) and CaMKII phosphorylation (F) for the excitatory neuron. Red stars indicate the default parameter combination. Note the smooth, monotonic gradients in contrast to the bifurcation structure observed for β_{NMDA} variation alone (cf. main text Figures 3–5).

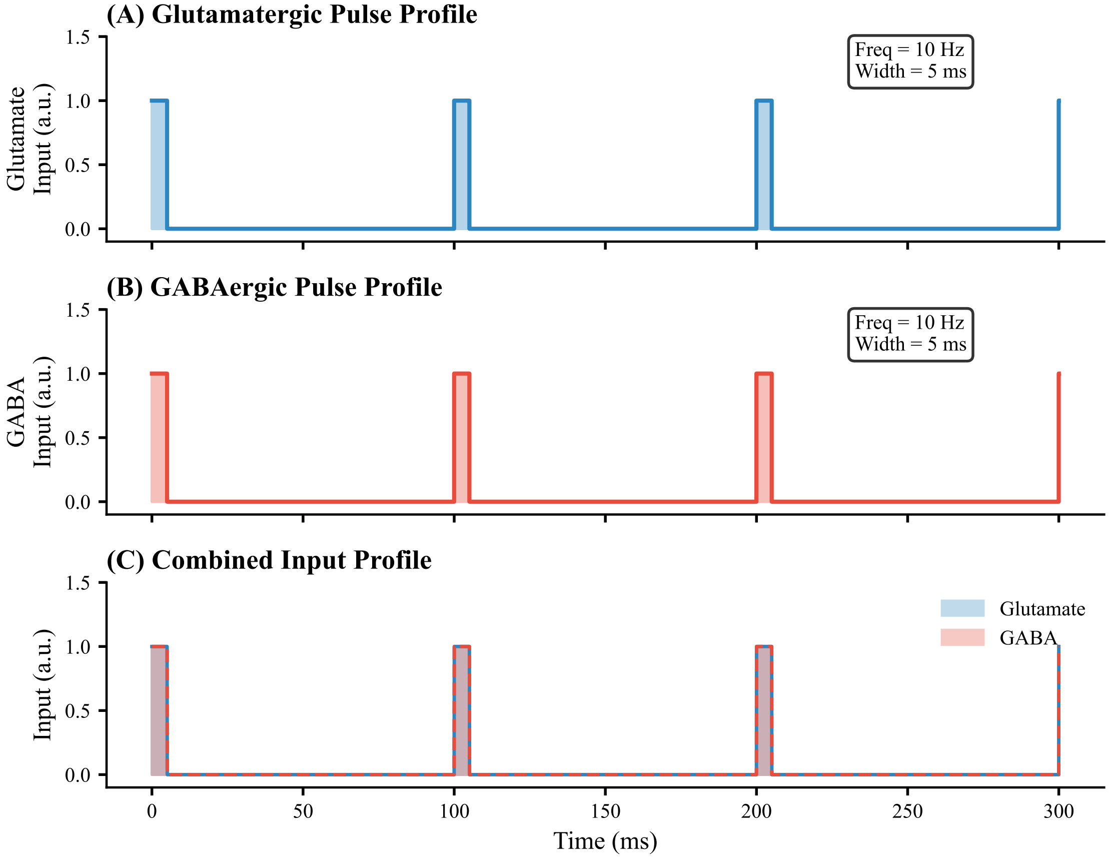

Figure S4: **Synaptic input pulse profiles.** (A) Glutamatergic rectangular pulse train (10 Hz, 5 ms pulse width, 1 μM amplitude) driving AMPA and NMDA receptor activation. (B) GABAergic pulse train with identical temporal parameters driving $GABA_A$ receptor activation. (C) Combined input profile showing simultaneous glutamatergic and GABAergic stimulation.

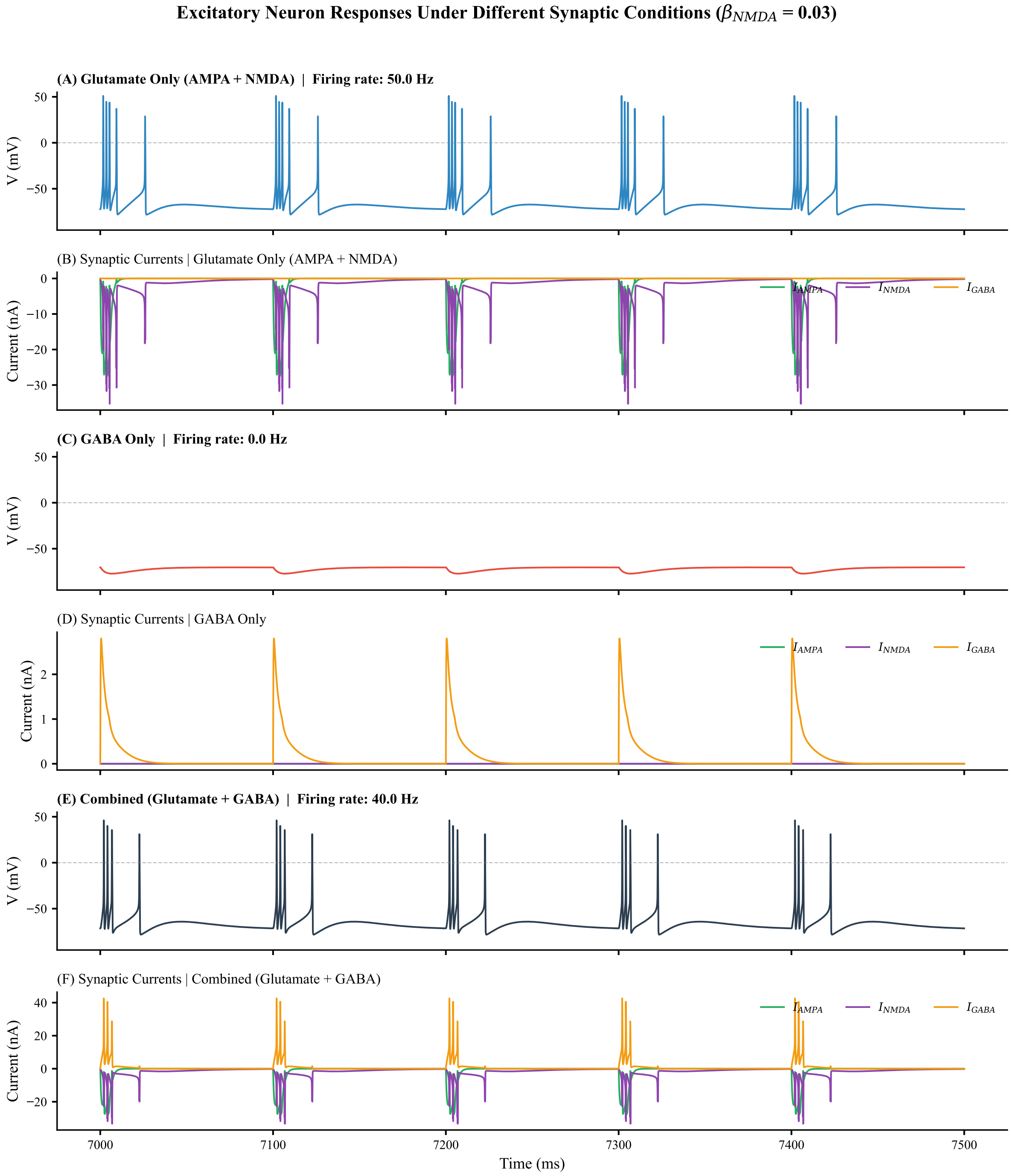

Figure S5: **Excitatory (pyramidal) neuron responses under separate and combined synaptic conditions** ($\beta_{\mathrm{NMDA}} = 0.03\ \mathrm{ms}^{-1}$). (A–B) Glutamate only (AMPA + NMDA): robust firing at 50 Hz with large excitatory synaptic currents. (C–D) GABA only: pure inhibitory input is insufficient to generate spikes (0 Hz firing rate), producing only subthreshold hyperpolarizing GABA currents. (E–F) Combined glutamate + GABA: GABA inhibition reduces firing rate from 50 Hz to 40 Hz and attenuates excitatory current amplitudes, demonstrating the modulatory role of GABAergic input on excitatory neuron dynamics.

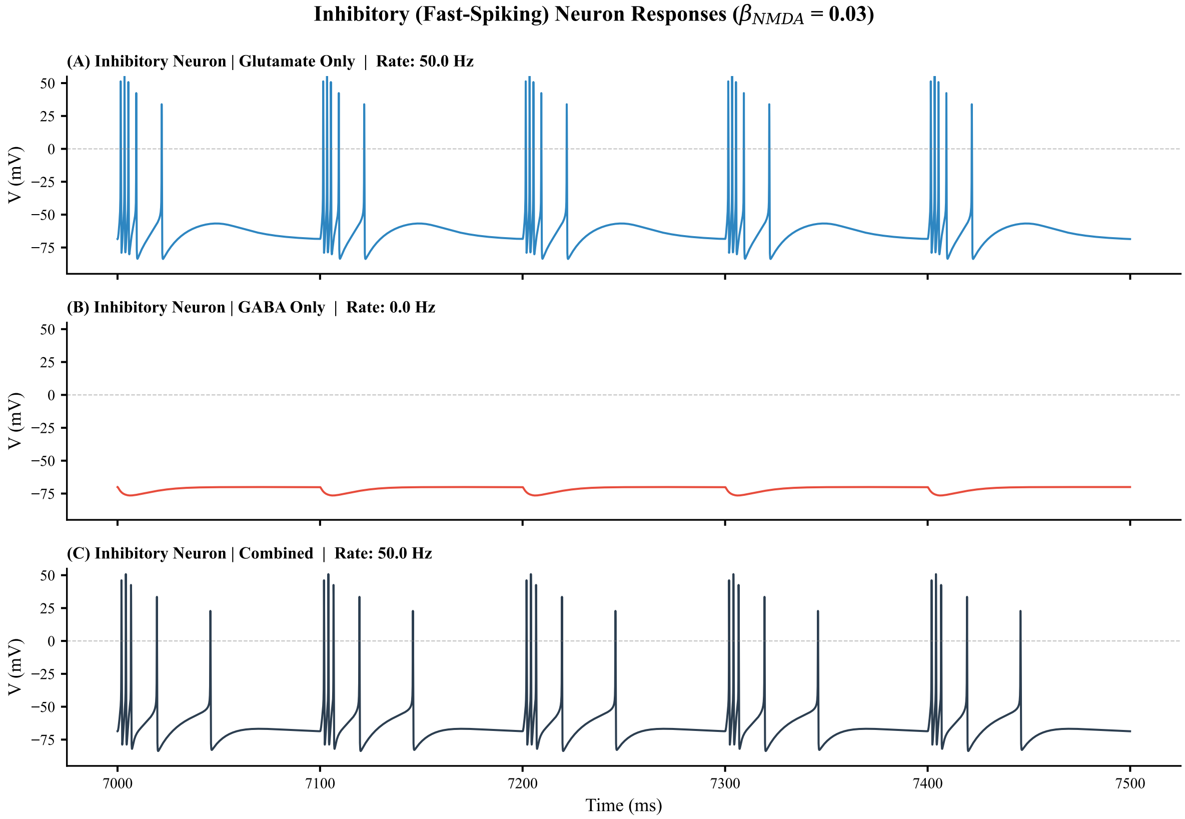

Figure S6: **Inhibitory (fast-spiking) neuron responses under separate and combined synaptic conditions** ($\beta_{\text{NMDA}} = 0.03$ ms^{-1}). (A) Glutamate only: the fast-spiking neuron exhibits burst-like patterns at 50 Hz with high-frequency spike clusters. (B) GABA only: as with the excitatory neuron, pure inhibitory input does not evoke spikes (0 Hz). (C) Combined: GABA does not substantially reduce the inhibitory neuron firing rate (50 Hz), indicating that fast-spiking interneurons are more resistant to GABAergic modulation than pyramidal neurons. Compare with excitatory neuron (Figure S5) to observe the differential effects of identical synaptic inputs on excitatory versus inhibitory cell types.

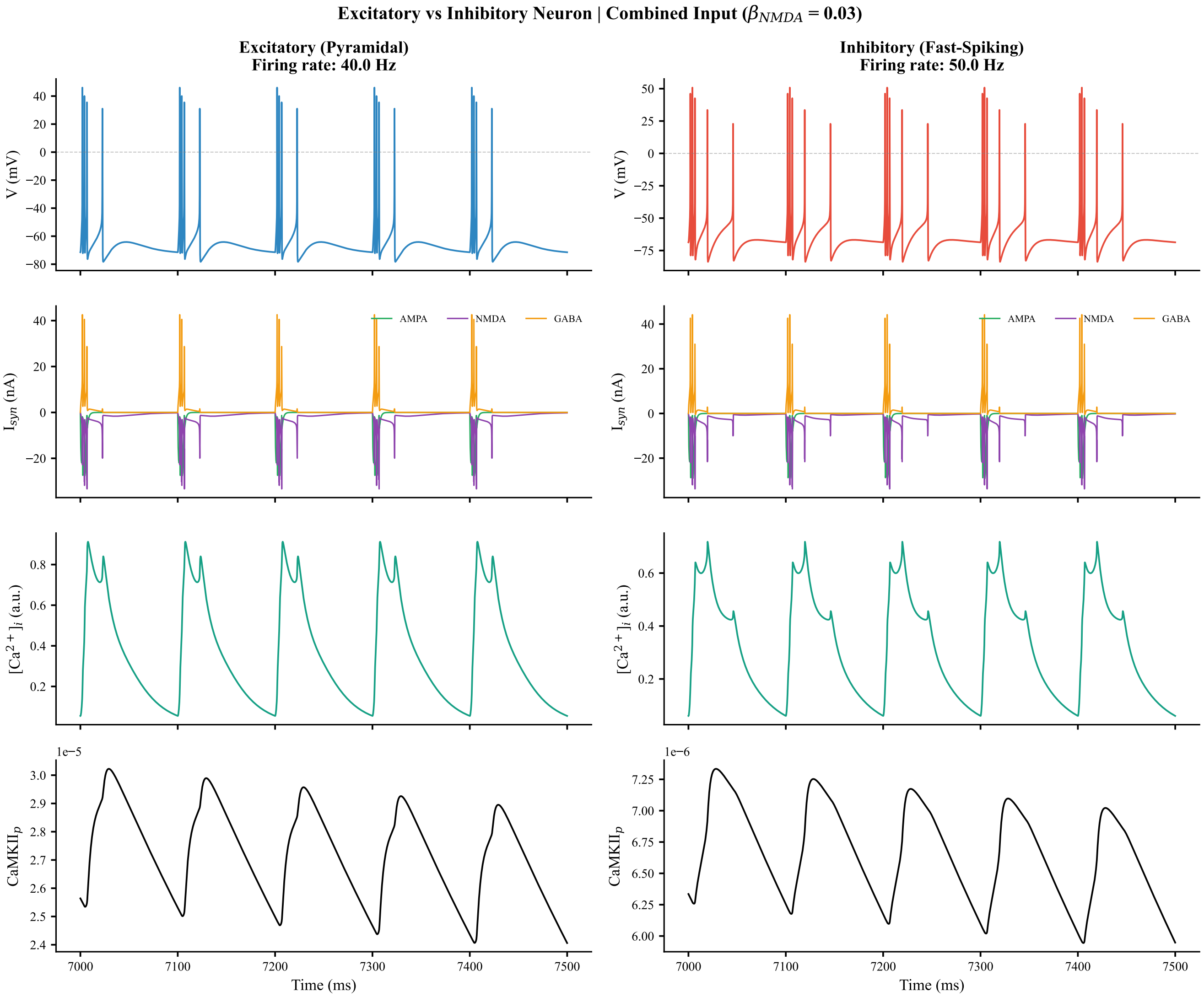

Figure S7: **Side-by-side comparison of excitatory and inhibitory neuron responses under combined glutamatergic and GABAergic input** ($\beta_{\text{NMDA}} = 0.03$ ms^{-1}). Four rows show: membrane potential (top), individual synaptic currents (I_{AMPA}, I_{NMDA}, I_{GABA}; second row), intracellular calcium transients (third row), and CaMKII phosphorylation dynamics (bottom). The excitatory neuron fires at 40 Hz while the inhibitory neuron fires at 50 Hz under combined input. The excitatory neuron exhibits larger calcium transients (peak ∼0.9 vs. ∼0.65 a.u.) and approximately 4-fold higher CaMKII phosphorylation (∼3×10^{-5} vs. ∼7×10^{-6}), reflecting the stronger NMDA-mediated calcium influx characteristic of pyramidal neurons.

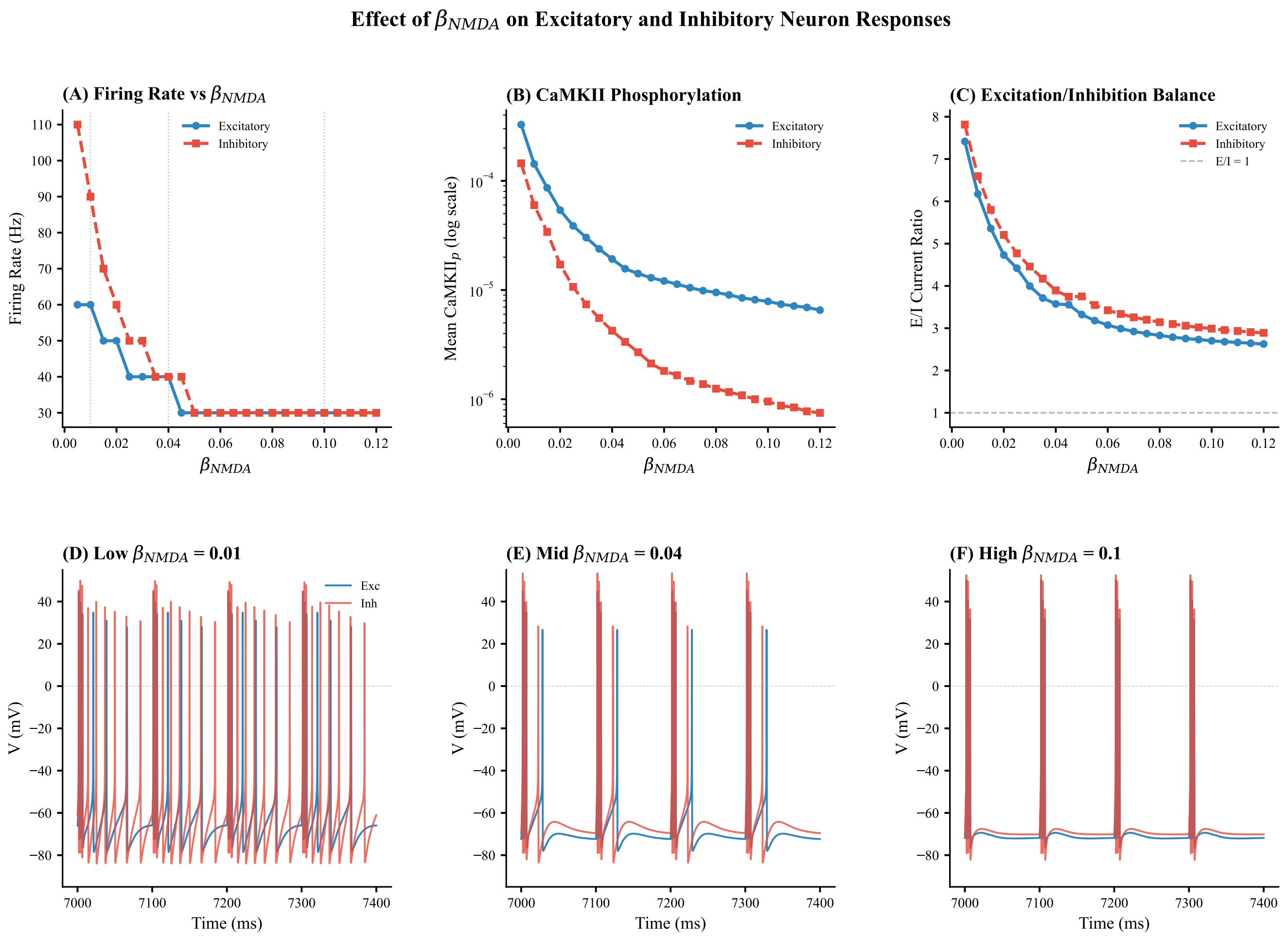

Figure S8: **Differential effects of β_{NMDA} on excitatory and inhibitory neurons.** (A) Firing rate vs. β_{NMDA} for both neuron types under combined input, showing the inhibitory neuron starts at higher rates ($\sim$110 Hz at low β_{NMDA}) and both converge to $\sim$30 Hz. Dashed vertical lines mark representative values shown in panels D–F. (B) CaMKII phosphorylation (log scale) decreases monotonically with increasing β_{NMDA} for both types, with excitatory neurons maintaining consistently higher phosphorylation. (C) Excitation/inhibition current ratio decreases from $\sim$8 to $\sim$3 across the β_{NMDA} range, remaining above the balanced state (E/I = 1) throughout. (D–F) Representative membrane potential traces at low (β_{NMDA} = 0.01), mid (0.04), and high (0.1) values, illustrating the transition from high-frequency irregular firing to sparse regular spiking.

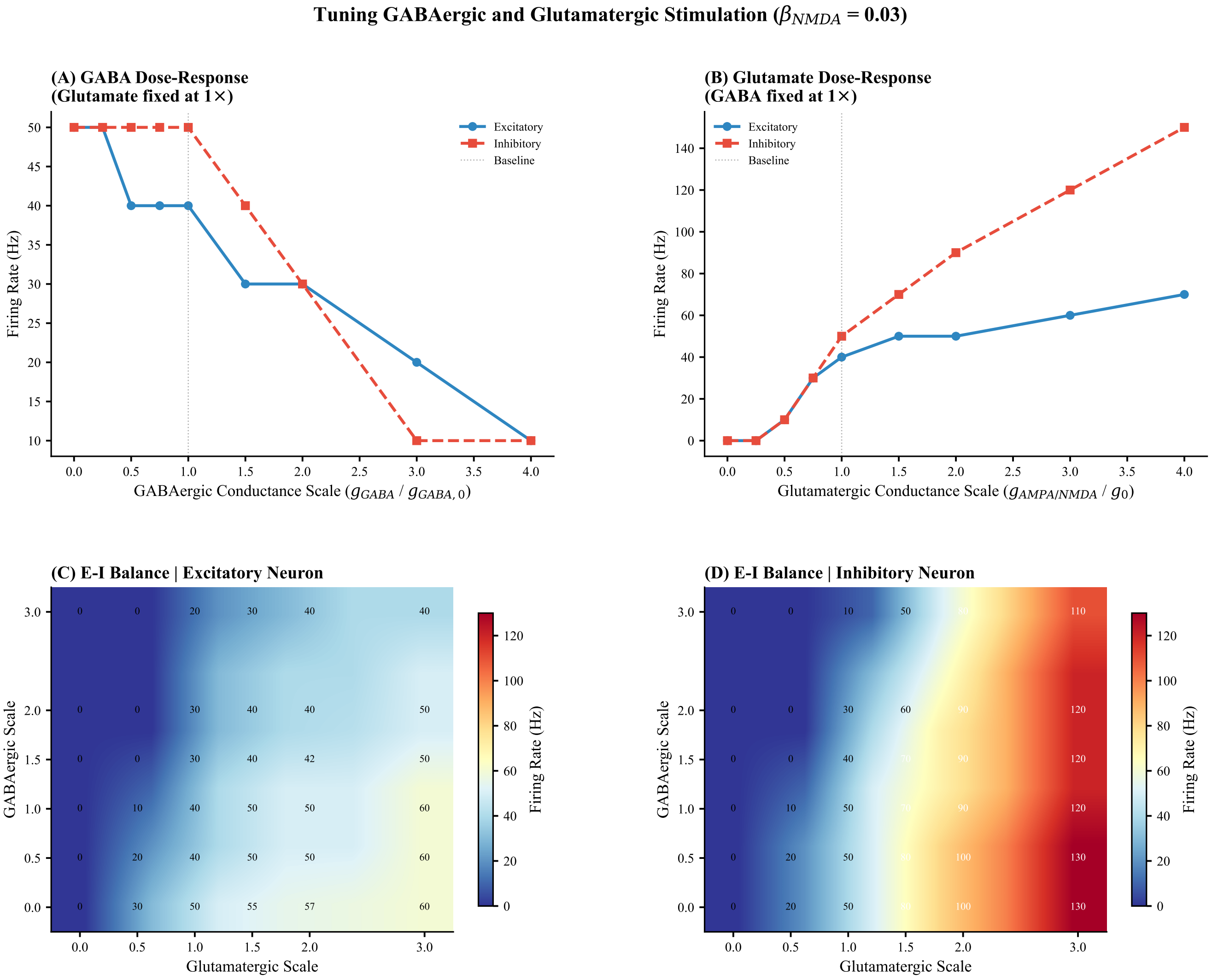

Figure S9: **Conductance tuning and excitation–inhibition balance** ($\beta_{\text{NMDA}} = 0.03\ \text{ms}^{-1}$). (A) GABA dose-response: scaling GABAergic conductance (at fixed glutamate) progressively reduces firing rate in both neuron types, with the excitatory neuron showing greater sensitivity to inhibition. (B) Glutamate dose-response: scaling glutamatergic conductance (at fixed GABA) increases firing rate, with the inhibitory neuron showing steeper gain (reaching ∼140 Hz at 4× baseline). (C–D) Two-dimensional heatmaps of firing rate across the full glutamatergic–GABAergic conductance scaling space for excitatory (C) and inhibitory (D) neurons. The diagonal gradient illustrates the competitive balance between excitatory and inhibitory drive, with the inhibitory neuron exhibiting a broader responsive region at high glutamatergic scale.

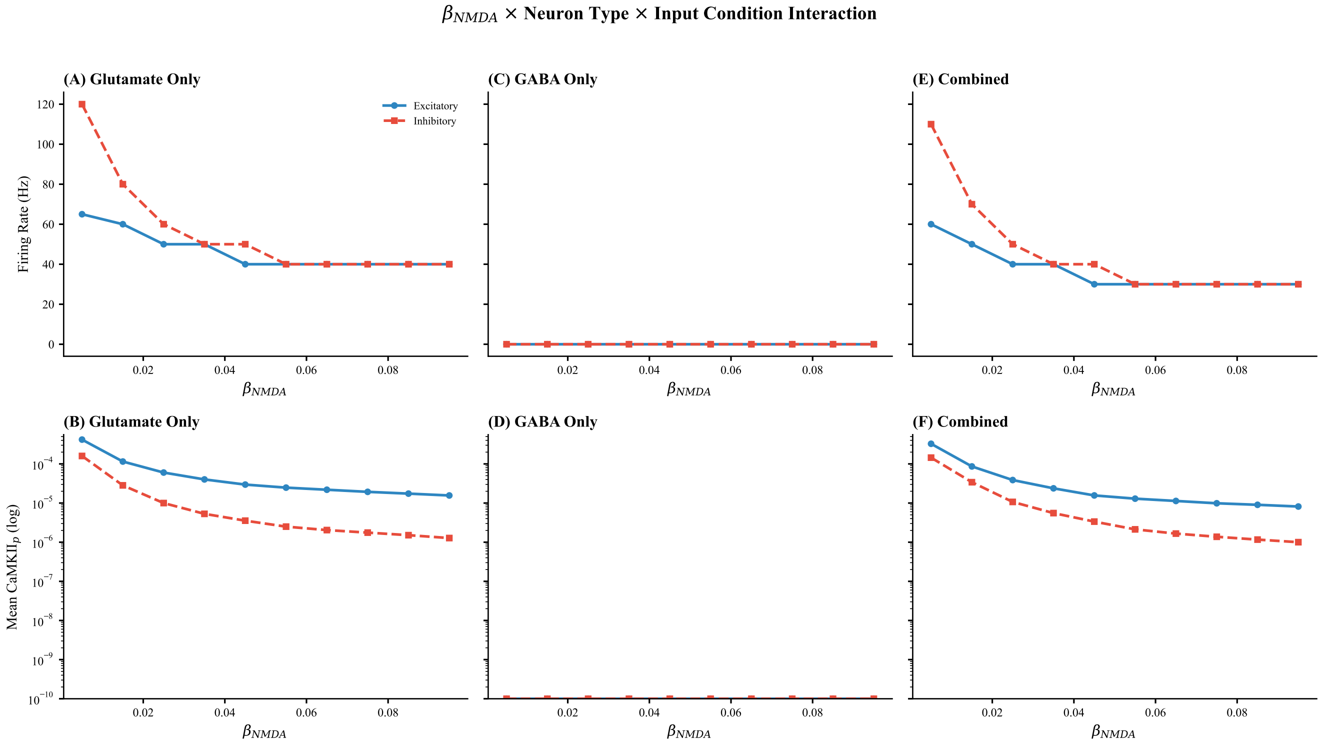

Figure S10: **Three-way interaction:** β_{NMDA} **× neuron type × input condition.** Top row: firing rate as a function of β_{NMDA} under glutamate only (A), GABA only (C), and combined input (E) for excitatory (blue) and inhibitory (red) neurons. Bottom row: corresponding mean CaMKII phosphorylation (log scale; B, D, F). Key findings: (A–B) Under glutamate only, both neuron types show decreasing firing rate and CaMKII with increasing β_{NMDA}, with the inhibitory neuron exhibiting higher firing rates but lower CaMKII. (C–D) GABA alone produces no spikes and negligible CaMKII in either type. (E–F) Combined input reduces overall firing rate and narrows the difference between neuron types, while CaMKII differences persist, indicating that the plasticity-relevant calcium–CaMKII pathway remains differentially activated even when firing rates converge.

References

György Buzsáki and Xiao-Jing Wang. Mechanisms of gamma oscillations. *Annual Review of Neuroscience*, 35:203–225, 2012. doi: 10.1146/annurev-neuro-062111-150444.

Fritjof Helmchen, Keiji Imoto, and Bert Sakmann. Ca2+ buffering and action potential-evoked Ca2+ signaling in dendrites of pyramidal neurons. *Biophysical Journal*, 70(2):1069–1081, 1996. doi: 10.1016/S0006-3495(96)79653-4.

Jeffry S Isaacson and Massimo Scanziani. How inhibition shapes cortical activity. *Neuron*, 72(2):231–243, 2011. doi: 10.1016/j.neuron.2011.09.027.

Ole Jensen and Ali Mazaheri. Shaping functional architecture by oscillatory alpha activity: gating by inhibition. *Frontiers in Human Neuroscience*, 4:186, 2010. doi: 10.3389/fnhum.2010.00186.

Björn M Kampa, John Clements, Peter Jonas, and Greg J Stuart. Kinetics of Mg2+ unblock of NMDA receptors: implications for spike-timing dependent synaptic plasticity. *The Journal of Physiology*, 556 (2):337–345, 2004. doi: 10.1113/jphysiol.2003.058842.

Henry Markram, Joachim Lübke, Michael Frotscher, and Bert Sakmann. Regulation of synaptic efficacy by coincidence of postsynaptic aps and epsps. *Science*, 275(5297):213–215, 1997. doi: 10.1126/science.275.5297.213.

Donghoon Shin, Kayla Peelman, Anthony D Lien, Joseph Del Rosario, and Bilal Haider. Narrowband gamma oscillations propagate and synchronize throughout the mouse thalamocortical visual system. *Neuron*, 111(7):1076–1085.e8, 2023. doi: 10.1016/j.neuron.2023.03.006.

Joshua H Siegle, Xiaoxuan Jia, Séverine Durand, Sam Gale, Charles Bennett, Nika Graddis, Greggory Heller, Thomas K Ramirez, Hyojin Choi, Jeffrey A Luviano, et al. Survey of spiking in the mouse visual system reveals functional hierarchy. *Nature*, 592(7852):86–92, 2021. doi: 10.1038/s41586-020-03171-x.

Mariana Vargas-Caballero and Hugh PC Robinson. Fast and slow voltage-dependent dynamics of magnesium block in the NMDA receptor: the asymmetric trapping block model. *Journal of Neuroscience*, 24(27):6171–6180, 2004. doi: 10.1523/JNEUROSCI.1380-04.2004.
